# Self‐Rated Health Status and Risk of Ischemic Heart Disease in the China Kadoorie Biobank Study: A Population‐Based Cohort Study

**DOI:** 10.1161/JAHA.117.006595

**Published:** 2017-09-22

**Authors:** Wenhong Dong, Xiong‐Fei Pan, Canqing Yu, Jun Lv, Yu Guo, Zheng Bian, Ling Yang, Yiping Chen, Tangchun Wu, Zhengming Chen, An Pan, Liming Li, the China Kadoorie Biobank Collaborative Group, Junshi Chen, Zhengming Chen, Rory Collins, Richard Peto, Daniel Avery, Ruth Boxall, Bennett Derrick, Yumei Chang, Robert Clarke, Huaidong Du, Simon Gilbert, Alex Hacker, Mike Hill, Michael Holmes, Andri Iona, Christiana Kartsonaki, Rene Kerosi, Ling Kong, Om Kurmi, Garry Lancaster, Sarah Lewington, Kuang Lin, John McDonnell, Iona Millwood, Qunhua Nie, Jayakrishnan Radhakrishnan, Sajjad Rafiq, Paul Ryder, Sam Sansome, Dan Schmidt, Paul Sherliker, Rajani Sohoni, Becky Stevens, Iain Turnbull, Robin Walters, Jenny Wang, Lin Wang, Neil Wright, Ling Yang, Xiaoming Yang, Zheng Bian, Yu Guo, Xiao Han, Can Hou, Jun Lv, Pei Pei, Yunlong Tan, Canqing Yu, Zengchang Pang, Ruqin Gao, Shanpeng Li, Shaojie Wang, Yongmei Liu, Ranran Du, Yajing Zang, Liang Cheng, Xiaocao Tian, Hua Zhang, Yaoming Zhai, Feng Ning, Xiaohui Sun, Feifei Li, Silu Lv, Junzheng Wang, Wei Hou, Mingyuan Zeng, Ge Jiang, Xue Zhou, Liqiu Yang, Hui He, Bo Yu, Yanjie Li, Qinai Xu, Quan Kang, Ziyan Guo, Dan Wang, Ximin Hu, Hongmei Wang, Jinyan Chen, Yan Fu, Zhenwang Fu, Xiaohuan Wang, Min Weng, Zhendong Guo, Shukuan Wu, Yilei Li, Huimei Li, Zhifang Fu, Ming Wu, Yonglin Zhou, Jinyi Zhou, Ran Tao, Jie Yang, Jian Su, Fang liu, Jun Zhang, Yihe Hu, Yan Lu, Liangcai Ma, Aiyu Tang, Shuo Zhang, Jianrong Jin, Jingchao Liu, Zhenzhu Tang, Naying Chen, Ying Huang, Mingqiang Li, Jinhuai Meng, Rong Pan, Qilian Jiang, Jian Lan, Yun Liu, Liuping Wei, Liyuan Zhou, Ningyu Chen, Ping Wang, Fanwen Meng, Yulu Qin, Sisi Wang, Xianping Wu, Ningmei Zhang, Xiaofang Chen, Weiwei Zhou, Guojin Luo, Jianguo Li, Xiaofang Chen, Xunfu Zhong, Jiaqiu Liu, Qiang Sun, Pengfei Ge, Xiaolan Ren, Caixia Dong, Hui Zhang, Enke Mao, Xiaoping Wang, Tao Wang, Xi zhang, Ding Zhang, Gang Zhou, Shixian Feng, Liang Chang, Lei Fan, Yulian Gao, Tianyou He, Huarong Sun, Pan He, Chen Hu, Xukui Zhang, Huifang Wu, Pan He, Min Yu, Ruying Hu, Hao Wang, Yijian Qian, Chunmei Wang, Kaixu Xie, Lingli Chen, Yidan Zhang, Dongxia Pan, Qijun Gu, Yuelong Huang, Biyun Chen, Li Yin, Huilin Liu, Zhongxi Fu, Qiaohua Xu, Xin Xu, Hao Zhang, Huajun Long, Xianzhi Li, Libo Zhang, Zhe Qiu

**Affiliations:** ^1^ Ministry of Education Key Laboratory of Environment and Health, and State Key Laboratory of Environmental Health (Incubating) School of Public Health Tongji Medical College Huazhong University of Science and Technology Wuhan China; ^2^ Department of Epidemiology and Biostatistics School of Public Health Beijing University Beijing China; ^3^ Chinese Academy of Medical Sciences Beijing China; ^4^ Clinical Trial Service Unit & Epidemiological Studies Unit (CTSU) Nuffield Department of Population Health University of Oxford Oxford United Kingdom

**Keywords:** Chinese, cohort study, ischemic heart disease, self‐rated health status, Cardiovascular Disease, Epidemiology, Risk Factors, Coronary Artery Disease

## Abstract

**Background:**

Self‐rated health (SRH) is a strong predictor of mortality in different populations. However, the associations between SRH measures and risk of ischemic heart disease (IHD) have not been extensively explored, especially in a Chinese population.

**Methods and Results:**

More than 500 000 adults from 10 cities in China were followed from baseline (2004–2008) through December 31, 2013. Global and age‐comparative SRH were reported from baseline questionnaires. Incident IHD cases were identified through links to well‐established disease registry systems and the national health insurance system. During 3 423 542 person‐years of follow‐up, we identified 24 705 incident cases of IHD. In multivariable‐adjusted models, both global and age‐comparative SRH was significantly associated with incident IHD. Compared with excellent SRH, the hazard ratios for good, fair, and poor SRH were 1.02 (95% confidence interval [CI], 0.98–1.07), 1.32 (95% CI, 1.27–1.37), and 1.76 (95% CI, 1.68–1.85), respectively. Compared with better age‐comparative SRH, the hazard ratios for same and worse age‐comparative SRH were 1.23 (95% CI, 1.19–1.27) and 1.78 (95% CI, 1.70–1.86), respectively. The associations persisted in all subgroup analyses, although they were slightly modified by study location, education, and income levels.

**Conclusions:**

A simple questionnaire for self‐assessment of health status was significantly associated with incident IHD in Chinese adults. Individuals and healthcare providers can use SRH measures as a convenient tool for assessing future IHD risk.

**Clinical Perspective**

**What Is New?**

- Although self‐rated health status has been used as a predictor for cardiovascular and all‐cause mortality, its association with the risk of ischemic heart disease remains controversial.
- In this prospective megacohort of more than half a million Chinese, participants reporting poor global or worse age‐comparative self‐rated health had ≈75% higher risk of future ischemic heart disease (independent of multiple traditional risk factors for ischemic heart disease).
- The magnitude of association was different across study locations, education levels, and household income levels. Nevertheless, the associations were robust and persisted in all subgroup and sensitivity analyses.

**What Are the Clinical Implications?**

- Although more research is warranted, physicians can use the 2 easily obtained self‐rated health measures as screening tools to identify individuals who may have higher risk of incident ischemic heart disease and to provide health education and therapies to improve cardiovascular care.

## Introduction

Ischemic heart disease (IHD), also known as coronary heart disease, is a leading cause of disease burden worldwide in terms of both years of life lost from IHD deaths and years of disability lived with nonfatal IHD.^1^ Despite a decrease in age‐standardized mortality of fatal and nonfatal IHD in high‐income countries since 1990, IHD remained as a leading cause of death between 1990 and 2013 in China.^2^ A total of 230 million adults suffered from IHD^3^ and 1.4 million people died from IHD in China in 2013.^4^ Demographic and social transitions including rural‐to‐urban migration, population growth, and aging were projected to further increase IHD incidence in China.^5^ Thus, prediction and early detection of IHD is of great importance for the prevention and control of IHD.

As a simple and relatively subjective index, self‐rated health (SRH) status has been used to predict cardiovascular mortality^6^, ^7^, ^8^ and all‐cause mortality^6^, ^7^, ^8^, ^9^, ^10^, ^11^, ^12^, ^13^ in different populations. The association between SRH and risk of incident IHD has been less frequently explored. In an early prospective study in Danish adults,^14^ a positive association was observed, whereas another study in Finnish men reported that the positive association vanished after adjustment for comorbid diseases, suggesting that perceived health levels mainly reflected underlying disease burden.^13^ The US Health and Retirement Study found that SRH predicted onset of major chronic diseases including IHD.^15^ However, only global SRH was examined in these studies, and other measures of SRH such as age‐comparative SRH were not studied previously. All 3 studies had small sample sizes (<5000) with limited statistical power for subgroup analyses. To the best of our knowledge, no study ever evaluated the predictive value of SRH for incident IHD in the Chinese population, even though SRH was found to predict mortality in Chinese adults.^11^, ^16^, ^17^

We aimed to investigate associations of global SRH and age‐comparative SRH with incident IHD using data from the China Kadoorie Biobank. Given the inconsistency and limitations of prior studies, we hypothesized that our study would support the utility of subjective health assessment for predicting IHD.

## Methods

### Study Population

The China Kadoorie Biobank study is a large population‐based prospective cohort study launched in China in 2004. This study was designed to investigate the influence of environmental factors, genetic variations, and their interactions on the morbidity and mortality of various chronic diseases. Participants were from 10 regions covering 5 rural counties (Gansu, Henan, Zhejiang, Sichuan, and Hunan provinces) and 5 urban cities (Harbin, Qingdao, Suzhou, Liuzhou, and Haikou); among these 10 regions, 4 are located in north China (Harbin, Qingdao, Gansu, and Henan), whereas the other 6 are in the south. The cohort design, sampling strategy, and baseline characteristics were reported elsewhere.^18^, ^19^ A total of 512 891 adult residents aged 30 to 79 years were enrolled between 2004 and 2008. Baseline information such as demographic characteristics; socioeconomic status (SES); personal behaviors; general health; family history; mental health; and, for women only, reproductive history was obtained by trained community‐ or village‐level health professionals via a computerized direct data‐entry system for the questionnaires. After the interview, various physical measurements such as height, weight, and blood pressure were undertaken for each participant, and a 10‐mL blood sample was collected.

We excluded 26 350 participants with prior history of IHD (n=15 472), rheumatic heart disease (n=938), stroke or transient ischemic attack (n=8884), or cancer (n=2577), as well as 2 persons with missing values for body mass index. A total of 486 541 participants (199 113 men and 287 428 women) were included in the final analysis. The study was approved by the ethics review committee of the Chinese Center for Disease Control and Prevention (Beijing, China) and the Oxford Tropical Research Ethics Committee, University of Oxford (UK). Written informed consent forms were obtained from all participants included in the study.

### Exposure Variables

Two questions were asked in baseline interviews to assess the SRH status of each participant: (1) How is your current general health status: excellent, good, fair, or poor? (2) How is your current health status compared with someone your own age: better, about the same, worse, or don't know? We considered the first question as global SRH and the second as age‐comparative SRH. Participants answering “don't know” for the second question (n=14 990, 3.08%) were excluded when examining the association between age‐comparative SRH and incident IHD. All participants answered the 2 questions, and no data were missing for the 2 assumed exposure variables.

### Outcome Variables

Information on incident IHD was regularly collected through linkage with the regional disease registry system and, through unique national identifiers, with an established national health insurance system, which records details of *International Classification of Diseases, 10th Revision* (ICD‐10) coded hospitalization information.^18^ The vital status of each participant was obtained by linkage to China's Disease Surveillance Points system, checked annually against local residential cards, health insurance records, and local street committees.^19^ Scanned copies of original disease reporting cards and official death certificates were obtained for each incident event, and new IHD cases were adjudicated by trained physicians blinded to the baseline exposure status and were coded as I20 to I25 (ICD‐10).

### Confounding Factors

The following demographic and socioeconomic characteristics were obtained in baseline interviews: age, sex (male, female), study location, marital status (married, widowed, separated or divorced, never married), education level (no formal education, primary, middle or high school, college/university or higher), annual household income (in Chinese yuan: <10 000 [<$1531], 10 000–19 999 [$1531–3062], 20 000–34 999 [$3063–5361], ≥35 000 [≥$5361]), and occupation (farmers, factory workers, professionals and managers, retirees, unemployed and others). Women were asked about their menopausal status (pre‐, peri‐, and postmenopause). All participants were also asked about their lifestyle behaviors, such as cigarette smoking (never, former, occasionally, and current smoker), alcohol consumption (never, former, occasionally, and weekly), sleep problems (yes or no), and physical activity level (calculated by adding up metabolic equivalent tasks for daily work or leisure activities). Eleven types of chronic comorbidities including tuberculosis, asthma, cirrhosis, chronic hepatitis, peptic ulcer, gall/bladder stone, kidney disease, fracture, rheumatoid arthritis, psychiatric disorder, and head injury were self‐reported at baseline. Participants were also asked about diabetes mellitus and hypertension, as well as family history of heart disease. Prevalent diabetes mellitus was defined as self‐reported diagnosis of diabetes mellitus, use of antidiabetic medications, or fasting plasma glucose ≥7.0 mmol/L.^20^ Prevalent hypertension was defined as measured systolic blood pressure ≥140 mm Hg and/or diastolic blood pressure ≥90 mm Hg, self‐reported diagnosis of hypertension, or use of antihypertensive medication.^21^ Past‐year major depression was assessed using the Chinese version of the computerized Composite International Diagnostic Inventory–Short Form; a detailed description of the assessment procedure was given elsewhere.^22^ Body height and weight were measured by trained staff, and body mass index was calculated as weight in kilograms divided by the square of height in meters.

### Statistical Analyses

Baseline characteristics by global SRH categories and age‐comparative SRH groups were compared using ANOVA and χ^2^ tests for continuous and categorical variables, respectively. Person‐years were calculated by entry into the study until the onset of IHD; death; loss to follow‐up; or December 31, 2013, whichever came first. Cox proportional hazards regression models were used to estimate hazard ratios (HRs) and 95% confidence intervals (CIs) for associations between 2 measures of SRH and incident IHD, with stratification according to age at baseline (at 5‐year intervals), sex, and study location. We adjusted for putative IHD risk factors and potential confounders in sequential steps: Model 1 adjusted for sociodemographic factors (continuous age, marital status, education level, household income, occupation, and menopausal status); model 2 further adjusted for lifestyle factors (sleep problems, cigarette smoking, alcohol consumption, and physical activity level), body mass index, and family history of heart disease; and model 3 also adjusted for baseline major depression, diabetes mellitus, hypertension, and presence of other comorbidities (yes if participant has any of the eleven chronic diseases and no if participant has none of the eleven chronic diseases.

Global SRH and age‐comparative SRH were analyzed separately as exposures and were incorporated in the same model to explore whether they were independent of each other. To compare the predictive power of the 2 exposures, Harrell's C statistic was calculated in both model 3 plus global SRH and model 3 plus age‐comparative SRH. Stratified analyses were performed by age (30–59, 60–69, and ≥70 years), sex, administrative regions (urban and rural), geographical regions (north and south), study location (10 locations), education level (no formal education, primary school, middle school or higher), household income (<10 000, 10 000–34 999, and ≥35 000 Chinese yuan), cigarette smoking (never, former, and current smoker), alcohol consumption (never, former, and current drinker), physical activity (low, moderate, high), body mass index (<18.5, 18.5–23.9, 24.0–27.9, and ≥28.0 kg/m^2^), hypertension, and diabetes mellitus. Tests for interaction were conducted by adding interaction terms in model 3. Sensitivity analyses were conducted to test the robustness of the association by excluding major depression, baseline comorbidities (diabetes mellitus, hypertension, and other comorbidities), and those who died or developed IHD in the first 2 years of follow‐up. Sensitivity analysis was also conducted by including those reporting “don't know” to the age‐comparative SRH question. All analysis was performed using SAS 9.3 (SAS Institute Inc), and 2‐sided *P*<0.05 was considered statistically significant.

## Results

The mean age (±SD) of participants was 51.0±10.5 years at baseline (Table 1). Among the 486 541 participants, 18.2% reported excellent SRH, 29.0% reported good SRH, 43.6% reported fair SRH, and 9.2% reported poor SRH. Compared with participants with excellent global SRH, those who reported poor SRH were older, poorer, and less educated; they had higher prevalence of sleep problems, preexisting major depression, diabetes mellitus, hypertension, and other comorbidities; and they were less likely to be physically active, current drinkers, and smokers, but they were more likely to be farmers, female, single, and underweight (all *P*<0.001).

**Table 1:** Characteristics of the China Kadoorie Biobank Study by Global Self‐Rated Health Status at Baseline^a^

Variables	Total, N (%)	Global Self‐Rated Health, N (%)			
		Excellent	Good	Fair	Poor
N	486 541 (100)	88 340 (18.2)	141 022 (29.0)	212 158 (43.6)	45 021 (9.2)
Age, y^b^	51.0 (10.5)	49.4 (10.4)	50.4 (10.3)	51.8 (10.6)	52.7 (10.7)
Sex					
Male	199 113 (40.9)	41 050 (46.5)	60 002 (42.5)	82 715 (39.0)	15 346 (34.1)
Female	287 428 (59.1)	47 290 (53.5)	81 020 (57.5)	129 443 (61.0)	29 675 (65.9)
Marital status					
Married	44 228 (90.9)	81 482 (92.2)	130 083 (92.2)	191 112 (90.1)	39 551 (87.9)
Widowed	33 081 (6.8)	4648 (5.3)	8375 (6.0)	15 867 (7.5)	4191 (9.3)
Separated/divorced	7579 (1.6)	1611 (1.8)	1719 (1.2)	3431 (1.6)	818 (1.8)
Never married	3653 (0.7)	599 (0.7)	845 (0.6)	1748 (0.8)	461 (1.0)
Education level					
No formal school	90 829 (18.7)	13 334 (15.1)	28 823 (20.4)	37 529 (17.7)	11 143 (24.8)
Primary	156 407 (32.1)	23 148 (26.2)	45 874 (32.5)	71 239 (33.6)	16 146 (35.9)
Middle or high school	211 683 (43.5)	43 668 (49.4)	59 356 (42.1)	92 248 (43.5)	16 411 (36.4)
College/university or higher	27 622 (5.7)	8190 (9.3)	6969 (5.0)	11 142 (5.2)	1321 (2.9)
Household income, RMB					
<10 000	137 893 (28.3)	19 141 (21.7)	36 437 (25.8)	61 966 (29.2)	20 439 (45.4)
10 000–19 999	140 432 (28.9)	23 369 (26.4)	39 598 (28.0)	64 733 (30.5)	12 732 (28.3)
20 000–34 999	120 197 (24.7)	24 457 (27.7)	36 346 (25.8)	52 046 (24.5)	7348 (16.3)
≥35 000	88 019 (18.1)	21 373 (24.2)	28 731 (20.4)	33 413 (15.8)	4502 (10.0)
Occupation					
Farmers	208 754 (42.9)	28 356 (32.1)	65 913 (46.7)	91 496 (43.1)	22 989 (51.1)
Factory workers	71 331 (14.7)	18 606 (21.1)	22 618 (16.1)	26 435 (12.5)	3672 (8.2)
Professionals and managers	64 533 (13.3)	17 557 (19.9)	18 223 (12.9)	25 131 (11.8)	3622 (8.0)
Retired	72 156 (14.8)	13 307 (15.0)	17 102 (12.1)	35 868 (16.9)	5879 (13.0)
Unemployed and others	69 767 (14.3)	10 514 (11.9)	17 166 (12.2)	33 228 (15.7)	8859 (19.7)
Administrative region					
Rural	276 755 (56.9)	38 840 (44.0)	89 112 (63.2)	119 890 (56.5)	28 913 (64.2)
Urban	209 786 (43.1)	49 500 (56.0)	51 910 (36.8)	92 268 (43.5)	16 108 (35.8)
Geographic region					
North	190 992 (39.3)	42 784 (48.4)	53 293 (37.8)	77 851 (36.7)	17 064 (37.9)
South	295 549 (60.7)	45 556 (51.6)	87 729 (62.2)	134 307 (63.3)	27 957 (62.1)
Sleep problems (yes)	79 756 (16.4)	8295 (9.4)	18 301 (13.0)	38 238 (18.0)	14 922 (33.1)
Cigarette smoking					
Never	301 601 (62.0)	51 235 (58.0)	85 768 (60.8)	135 700 (64.0)	28 898 (64.2)
Former	26 807 (5.5)	4845 (5.5)	7618 (5.4)	11 392 (5.4)	2952 (6.6)
Occasionally	27 872 (5.7)	5329 (6.0)	7793 (5.5)	12 175 (5.7)	2575 (5.7)
Current	130 261 (26.8)	26 931 (30.5)	39 843 (28.3)	52 891 (24.9)	10 596 (23.5)
Alcohol consumption					
Never	222 161 (45.7)	35 164 (39.8)	63 648 (45.1)	100 758 (47.5)	22 591 (50.2)
Formerly	7751 (1.6)	851 (1.0)	1658 (1.2)	3692 (1.7)	1550 (3.4)
Occasionally	172 550 (35.4)	33 226 (37.6)	49 317 (35.0)	75 142 (35.4)	14 865 (33.0)
Weekly	84 079 (17.3)	19 099 (21.6)	26 339 (18.7)	32 566 (15.4)	6015 (13.4)
Physical activity, MET h/d^c^	18.1 (10.8–30.6)	19.7 (12.0–31.6)	20.2 (11.7–33.5)	16.8 (10.0–29.1)	15.5 (8.4–27.0)
Menopausal status (women only)					
Premenopause	127 325 (44.3)	25 360 (53.6)	38 646 (47.7)	53 125 (41.0)	10 194 (34.4)
Perimenopause	14 354 (5.0)	2271 (4.8)	4162 (5.1)	6489 (5.0)	1432 (4.8)
Postmenopause	145 749 (50.7)	19 659 (41.6)	38 212 (47.2)	69 829 (54.0)	18 049 (60.8)
BMI, kg/m^2^					
<18.5	21 428 (4.4)	2585 (2.9)	4751 (3.4)	10 602 (5.0)	3490 (7.8)
18.5–23.9	255 773 (52.6)	45 574 (51.6)	74 277 (52.7)	112 460 (53.0)	23 462 (52.1)
24.0–27.9	159 677 (32.8)	31 126 (35.2)	47 812 (33.9)	67 625 (31.9)	13 114 (29.1)
≥28.0	49 663 (10.2)	9055 (10.3)	14 182 (10.0)	21 471 (10.1)	4955 (11.0)
Family history of heart disease (yes)	15 379 (3.2)	2976 (3.4)	4171 (3.0)	6727 (3.2)	1505 (3.3)
Baseline major depression (yes)	2972 (0.6)	216 (0.2)	471 (0.3)	1328 (0.6)	957 (2.1)
Baseline diabetes mellitus (yes)	26 118 (5.4)	3143 (3.6)	5433 (3.9)	12 839 (6.1)	4703 (10.5)
Baseline hypertension (yes)	158 473 (32.6)	24 959 (28.3)	44 694 (31.7)	72 026 (34.0)	16 794 (37.3)
Other baseline comorbidities (yes)	229 350 (47.1)	35 715 (40.4)	61 852 (43.9)	104 243 (49.1)	27 540 (61.2)

BMI indicates body mass index; MET, metabolic equivalent; RMB, Chinese yuan renminbi.

a Two‐sided *P* values were derived from ANOVA for continuous variables and from the χ^2^ test for categorical variables, all *P*<0.001.

b Data are shown as mean (SD).

c Data are shown as median (25th–75th percentiles).

Most participants (63.5%) reported age‐comparative SRH as same, whereas 18.7% reported better, 14.8% reported worse, another 3.1% reported “don't know” (Table S1). Comparisons of characteristics between better and worse age‐comparative SRH were consistent with those between excellent and poor global SRH.

**Figure 1:**
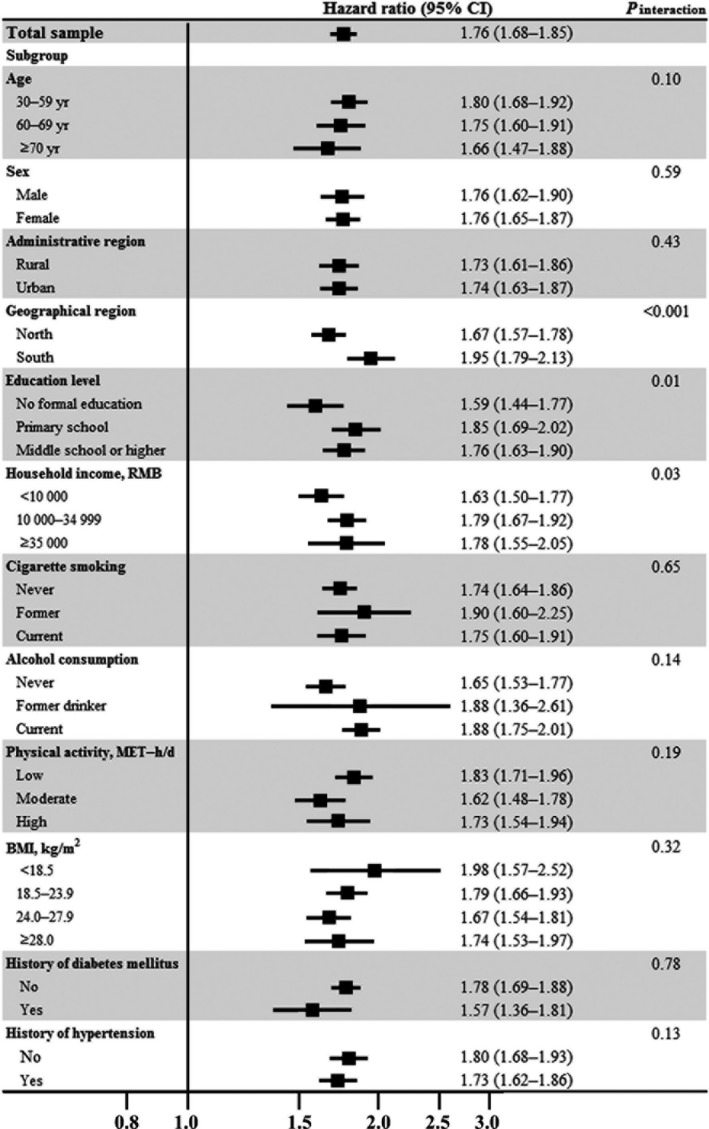
Poor vs excellent global self‐rated health and risk of ischemic heart disease in the China Kadoorie Biobank study: stratified analysis. Hazard ratios (95% CIs) were calculated after adjustment for age, marital status, household income, education level, occupation, menopausal status, sleep problems, alcohol consumption, cigarette smoking, physical activity, body mass index, family history of heart disease, baseline major depression, diabetes mellitus, hypertension, and other comorbidities, except for the stratified variable in the corresponding stratified analysis. BMI indicates body mass index; CI, confidence interval; MET, metabolic equivalent; RMB, Chinese yuan renminbi.

A total of 24 705 incident IHD cases were identified during 7.0±1.5 years of follow‐up. Absolute incidence rates according to global SRH categories were 6.0, 5.3, 8.2, and 11.0 incident IHD cases per 1000 person‐years for participants reporting excellent, good, fair, and poor global SRH, respectively. Relative to those reporting excellent baseline SRH, people with poor SRH were 1.96 times more likely to develop IHD after adjustment for age, sex, study location, marital status, education level, household income, occupation, and menopausal status (Table 2). The association was attenuated but remained significant (HR 1.76; 95% CI, 1.68–1.85) after further controlling for various cardiovascular risk factors such as baseline hypertension and diabetes mellitus. Although the association between SRH and incident IHD was modified by geographical region, education, and income (all *P* for interaction <0.05), significantly increased risk was observed across all subgroups in stratified analyses (Figure 1 and Table S2). No effect modifications by other covariates were observed.

**Table 2:** Risk of Ischemic Heart Disease According to 2 Measures of Self‐Rated Health Status

	Cases/person‐years	Model 1^a^	Model 2^b^	Model 3^c^
		HR (95% CI)	HR (95% CI)	HR (95% CI)
Global self‐rated health				
Excellent	3738/624 346	1.00	1.00	1.00
Good	5405/1 012 118	1.04 (1.00–1.08)	1.04 (0.99–1.08)	1.02 (0.98–1.07)
Fair	12 148/1 475 816	1.38 (1.33–1.44)	1.37 (1.31–1.42)	1.32 (1.27–1.37)
Poor	3414/311 262	1.96 (1.87–2.06)	1.90 (1.81–1.99)	1.76 (1.68–1.85)
Age‐comparative self‐rated health				
Better	4038/649 835	1.00	1.00	1.00
Same	14 939/2 172 181	1.27 (1.22–1.31)	1.26 (1.21–1.31)	1.23 (1.19–1.27)
Worse	5048/501 051	1.95 (1.87–2.04)	1.90 (1.82–1.99)	1.78 (1.70–1.86)

a Model 1: adjusted for age, marital status, education level, household income, occupation, and menopausal status.

b Model 2: model 1 plus sleep problems, cigarette smoking, alcohol consumption, physical activity, body mass index, and family history of heart disease.

c Model 3: model 2 plus baseline major depression, diabetes mellitus, hypertension, and other comorbidities.

Substantial variations in different study locations were found for the association between SRH and IHD risk: The HRs comparing poor with excellent SRH ranged from 1.24 (95% CI, 1.09–1.41) in Gansu residents to 2.77 (95% CI, 2.06–3.73) in Sichuan residents (Figure S1).

**Figure 2:**
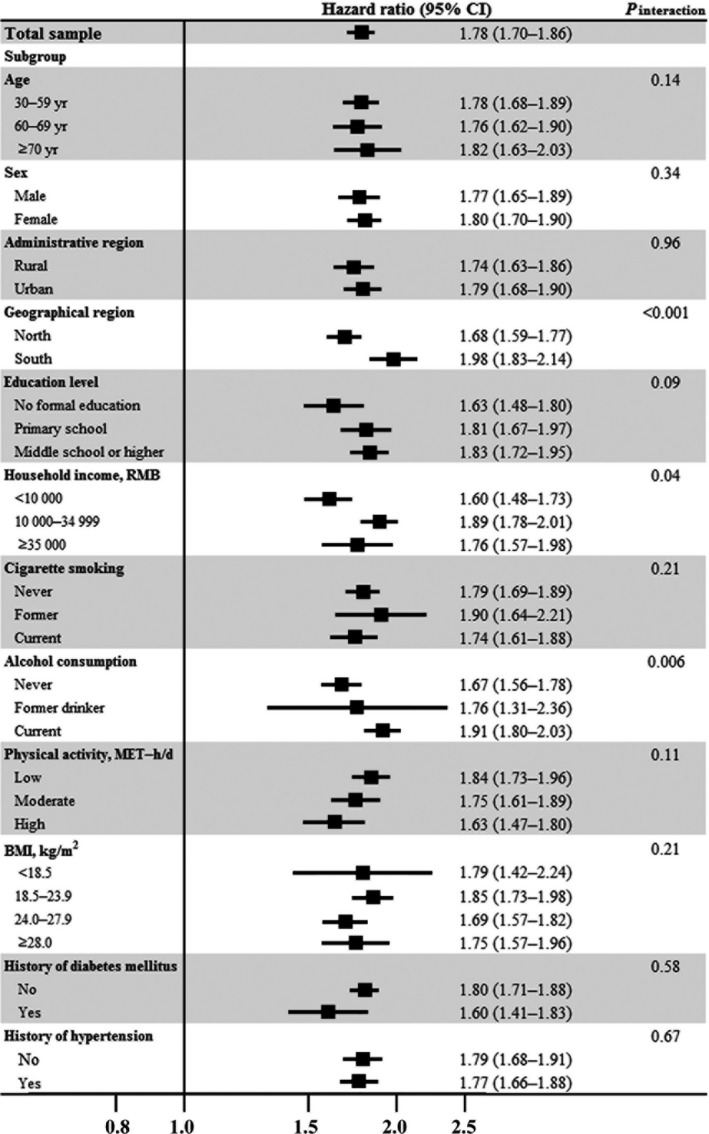
Worse vs better age‐comparative self‐rated health and risk of ischemic heart disease in the China Kadoorie Biobank study: stratified analysis. Hazard ratios (95% confidence intervals) were calculated after adjustment of age, marital status, household income, education level, occupation, menopausal status, sleep problems, alcohol consumption, cigarette smoking, physical activity, body mass index, family history of heart disease, baseline major depression, diabetes melllitus, hypertension, and other comorbidities, except for the stratified variable in the corresponding stratified analysis. BMI indicates body mass index; CI, confidence interval; MET, metabolic equivalent; RMB, Chinese yuan renminbi.

The absolute incidence rates according to age‐comparative SRH groups were 6.2, 6.9, and 10.1 incident IHD cases per 1000 person‐years for participants reporting better, same, and worse age‐comparative SRH, respectively. Compared with participants who reported better age‐comparative SRH status, participants reporting worse age‐comparative SRH had an 78% increased risk of developing IHD (HR: 1.78; 95% CI, 1.70–1.86; Table 2). The association merely changed in sensitivity analyses including those answering “don't know” (Table S3). Participants from north China with lower income showed a relatively weaker association (all *P*<0.05 for interaction), whereas the association was stronger among current drinkers versus never drinkers (*P*<0.001 for interaction; Figure 2 and Table S4). Effect modification by other covariates was not observed. When age‐comparative SRH and global SRH were incorporated into the same model, their effect sizes were attenuated, but both remained independently associated with incident IHD (Table S5). The distribution of global SRH by age‐comparative SRH was shown in Table S6. The weighted κ value of the consistency test for the 2 exposures was 0.29 (95% CI, 0.28–0.29), suggesting that they may provide overlapping information about health but are not interchangeable. The Harrel's C statistic values were both 0.77 in the final model with the 2 measures separately (*P*=0.39 comparing the 2 models).

Substantial variations were observed in the association among different regions, and the HR for IHD ranged from 1.19 (95% CI, 1.06–1.33) in Gansu residents to 2.92 (95% CI, 2.16–3.96) in Zhejiang residents when comparing worse versus better age‐comparative SRH (Figure S2).

After excluding those with major depression or baseline comorbidities or those who died or developed IHD in the first 2 years of follow‐up, associations between both global and age‐comparative SRH and risk of IHD merely changed (Table S7).

## Discussion

In this large population‐based prospective study, we found that both global and age‐comparative SRH was associated with the risk of developing IHD in Chinese adults. The associations were independent of multiple well‐established cardiovascular risk factors and might be modified by study location, education level, and household income.

Although SRH was considered a strong predictor of cardiovascular mortality^6^, ^7^, ^8^ and all‐cause mortality,^6^, ^7^, ^8^, ^9^, ^10^, ^11^, ^12^, ^13^ its utility for predicting risk of incident IHD has rarely been examined. In a Danish cohort of 1052 men and women,^14^ Moller et al first reported that poor or miserable SRH was associated with a substantially higher risk of IHD; however, the sample size was small, with only 50 incident cases during 16 years of follow‐up. In study of US adults aged 51 to 61 years,^15^ SRH was a significant predictor of new‐onset IHD, stroke, and other major chronic diseases except cancer. In this study, 691 incident IHD cases in 4770 participants were identified over a maximum of 16 years’ follow‐up. Another study among 2682 men aged 42 to 60 years in eastern Finland also reported a significant association between perceived health status and risk of myocardial infarction^13^; however, the association was attenuated to null after adjustment for baseline comorbidities (including symptomatic and asymptomatic coronary heart disease, stroke, diabetes mellitus, cancer, etc). The study followed participants for an average of 5.8 years and identified 205 incident myocardial infarction cases, but baseline cardiovascular diseases were not excluded. These studies suffered from limitations such as small sample size, single‐sex participants, and small number of incident cases,^13^, ^14^, ^15^ and their findings were largely inconsistent. Our study is thus the largest prospective study on this topic and the first in an Asian population. We used data from an ongoing Chinese megacohort study with participants who had different SES, lifestyles, and disease profiles. Consequently, we believe that our study provides compelling evidence of the association between SRH and incident IHD.

Because of the large sample size, we were able to explore associations in different subgroups, which was not possible in the previous 3 studies of the association between SRH and incident IHD.^13^, ^14^, ^15^ We found that education and income, 2 commonly used indicators of SES, may modify the associations among global SRH, age‐comparative SRH, and IHD risk: The associations were stronger among those with higher SES. This finding is consistent with those from studies of the influence of SRH on mortality.^23^, ^24^ People with higher SES may better understand their underlying health risks or objective health conditions^25^ and may rate their health status more negatively when they are in a worse condition compared with their less educated or affluent counterparts.^23^ We also found that the associations were stronger in south versus north China and in urban versus rural areas; these results may be attributable to discrepancies in overall SES in different regions, although we controlled for education levels, household income, and occupation in the models. The associations varied substantially across 10 study locations, but the exact reasons are unknown. We suspect that this may be due to different economic development levels across study sites. Culture, lifestyle, environment, and disease patterns were all different in different cities,^26^, ^27^ which may also potentially influence both SRH and heart disease.^28^, ^29^, ^30^, ^31^, ^32^, ^33^ However, positive associations existed across all subgroups, and SRH may be a reliable measurement for predicting IHD risk.

In addition to global SRH, we also examined the association between age‐comparative SRH and risk of IHD. Some studies investigated the association of both measures of SRH with mortality,^6^, ^10^, ^12^, ^34^, ^35^, ^36^, ^37^ but very few included both SRH measures in one model.^12^, ^35^, ^36^ Consequently, it is difficult to directly identify which one is better for predicting cardiovascular mortality. Some studies argued that the association between age‐comparative SRH and mortality was stronger and more consistent than that between global SRH and mortality^6^, ^10^, ^34^ because age‐comparative SRH had an explicit reference frame and compared health status for the same age, and thus was semantically clearer than global SRH.^10^, ^34^ Some thought, however, that global SRH was a preferable measure in longitudinal studies with a wide age range^12^, ^37^ because the age‐comparative index tended to improve with age because of lower expectations for health among older adults, and thus reporting may be less reliable. Others contended that both measures were equally predictors of mortality and may not be comparable because different wording may capture different domains of health.^35^, ^36^ In a semantic study, investigators reported that global SRH was mainly connected to unspecific terms related with health such as *function, habits, youth*, and *diet*, whereas age‐comparative SRH additionally led to social comparison, performance, and personal achievement.^38^ In our study, we controlled age in all models, but associations between 2 measures and risk of IHD were statistically significant in overall analysis, and the associations remained strong in each age stratum for both measures in stratified analysis. In the models where both global SRH and age‐comparative SRH were included, the magnitude of associations was reduced but both remained significant. Because the Harrel's C statistics were not different for the 2 predictive models including either global or age‐comparative SRH, we believe both global SRH and age‐comparative SRH are equally and independently useful for predicting risk of IHD.

Several reasons might explain the association among global SRH, age‐comparative SRH, and risk of IHD. First, global SRH and age‐comparative SRH are integrative measurements based on an individual's objective health status such as diagnosed diseases and treatment,^33^, ^39^ mental health (eg, depression, anxiety, and stress),^32^, ^33^, ^39^ and physical dysfunction.^32^, ^40^ They fairly well reflect current overall health status of an individual and thus predict future disease events. Second, poor SRH was reported to be associated with chronic inflammation (reflected by high levels of interleukin 6 and C‐reactive protein)^41^, ^42^, ^43^ and abnormal autonomic nervous system function,^44^ which are involved in the development of IHD. Third, unknown or underreported reasons such as individual disposition, nonfatal but lasting or frequently occurring ailments, and negative perceptions may result in unpleasant bodily sensations that could be perceived but not detected by traditional medical procedures.^45^ This pathway was not commonly recognized and should be further investigated.

The strengths of our study include its prospective study design, large sample size, multiple stratified analyses, and investigation of 2 measures of SRH in 1 study and in 1 model. This study also has a few limitations. First, inaccurate reports of exposures due to poor understanding of the 2 questions might exist. In our study, participants were from 10 locations and had differences in age, SES levels, culture, and social environments that could influence understanding of the questions and selection of the answers. Second, objective health status may confound the associations between SRH and IHD risk but might not be adequately adjusted in analyses. In one study,^13^ the association between SRH and myocardial infarction was attenuated to null when objective health conditions were added in the model. In our analyses, we excluded participants with baseline IHD, rheumatic heart disease, stroke, transient ischemic attack, and cancer and controlled for history of diabetes mellitus and hypertension in the models. We further excluded participants with major depression and comorbidities including diabetes mellitus, hypertension, and another 11 chronic diseases in the sensitivity analysis, and the associations remained unchanged. Third, there was a possibility of reverse causality bias. Participants may report poor global SRH or worse age‐comparative SRH at baseline because of undiagnosed cardiovascular disease; however, the positive associations between the 2 measures and IHD persisted even after we excluded those who died or developed IHD in the first 2 years after recruitment. Fourth, despite our efforts to control potential confounders, residual confounding might still be possible because of influence from factors such as air pollution, stress, or local culture. These might need to be controlled in future studies. Last, because all participants were Chinese, the results may not be generalized to other ethnicity groups; further investigations in other populations are warranted.

## Conclusions

Both global SRH and age‐comparative SRH were sensitive predictors of IHD risk in China. Our findings suggest that the 2 SRH measures may provide additional value on top of traditional clinical measurements and well‐established risk factors of IHD. They may be useful as screening tools in resource‐limited settings. Given the limited number of high‐quality studies on this specific topic, whether global SRH and age‐comparative SRH can be applied in IHD risk assessment model or whether they can be used as screening tools in community and clinical care deserve further investigation.

## Appendix

### Members of the China Kadoorie Biobank Collaborative Group

International Steering Committee: Junshi Chen, Zhengming Chen (PI), Rory Collins, Liming Li (PI), Richard Peto. International Coordinating Center, Oxford: Daniel Avery, Ruth Boxall, Derrick Bennett, Yumei Chang, Yiping Chen, Zhengming Chen, Robert Clarke, Huaidong Du, Simon Gilbert, Alex Hacker, Mike Hill, Michael Holmes, Andri Iona, Christiana Kartsonaki; Rene Kerosi, Ling Kong, Om Kurmi, Garry Lancaster, Sarah Lewington, Kuang Lin, John McDonnell, Iona Millwood, Qunhua Nie, Jayakrishnan Radhakrishnan, Sajjad Rafiq, Paul Ryder, Sam Sansome, Dan Schmidt, Paul Sherliker, Rajani Sohoni, Becky Stevens, Iain Turnbull, Robin Walters, Jenny Wang, Lin Wang, Neil Wright, Ling Yang, Xiaoming Yang. National Coordinating Center, Beijing: Zheng Bian, Yu Guo, Xiao Han, Can Hou, Jun Lv, Pei Pei, Yunlong Tan, Canqing Yu. 10 Regional Coordinating Centers: Qingdao Center for Disease Control and Prevention (CDC): Zengchang Pang, Ruqin Gao, Shanpeng Li, Shaojie Wang, Yongmei Liu, Ranran Du, Yajing Zang, Liang Cheng, Xiaocao Tian, Hua Zhang, Yaoming Zhai, Feng Ning, Xiaohui Sun, Feifei Li. Licang CDC: Silu Lv, Junzheng Wang, Wei Hou. Heilongjiang Provincial CDC: Mingyuan Zeng, Ge Jiang, Xue Zhou. Nangang CDC: Liqiu Yang, Hui He, Bo Yu, Yanjie Li, Qinai Xu,Quan Kang, Ziyan Guo. Hainan Provincial CDC: Dan Wang, Ximin Hu, Hongmei Wang, Jinyan Chen, Yan Fu, Zhenwang Fu, Xiaohuan Wang. Meilan CDC: Min Weng, Zhendong Guo, Shukuan Wu, Yilei Li, Huimei Li, Zhifang Fu. Jiangsu Provincial CDC: Ming Wu, Yonglin Zhou, Jinyi Zhou, Ran Tao, Jie Yang, Jian Su. Suzhou CDC: Fang liu, Jun Zhang, Yihe Hu, Yan Lu, Liangcai Ma, Aiyu Tang, Shuo Zhang, Jianrong Jin, Jingchao Liu. Guangxi Provincial CDC: Zhenzhu Tang, Naying Chen, Ying Huang. Liuzhou CDC: Mingqiang Li, Jinhuai Meng, Rong Pan, Qilian Jiang, Jian Lan, Yun Liu, Liuping Wei, Liyuan Zhou, Ningyu Chen, Ping Wang, Fanwen Meng, Yulu Qin, Sisi Wang. Sichuan Provincial CDC: Xianping Wu, Ningmei Zhang, Xiaofang Chen, Weiwei Zhou. Pengzhou CDC: Guojin Luo, Jianguo Li, Xiaofang Chen, Xunfu Zhong, Jiaqiu Liu, Qiang Sun. Gansu Provincial CDC: Pengfei Ge, Xiaolan Ren, Caixia Dong. Maiji CDC: Hui Zhang, Enke Mao, Xiaoping Wang, Tao Wang, Xi zhang. Henan Provincial CDC: Ding Zhang, Gang Zhou, Shixian Feng, Liang Chang, Lei Fan. Huixian CDC: Yulian Gao, Tianyou He, Huarong Sun, Pan He, Chen Hu, Xukui Zhang, Huifang Wu, Pan He. Zhejiang Provincial CDC: Min Yu, Ruying Hu, Hao Wang. Tongxiang CDC: Yijian Qian, Chunmei Wang, Kaixu Xie, Lingli Chen, Yidan Zhang, Dongxia Pan, Qijun Gu. Hunan Provincial CDC: Yuelong Huang, Biyun Chen, Li Yin, Huilin Liu, Zhongxi Fu, Qiaohua Xu. Liuyang CDC: Xin Xu, Hao Zhang, Huajun Long, Xianzhi Li, Libo Zhang, Zhe Qiu.

## Sources of Funding

This work was supported by the National Natural Science Foundation of China (81390540, 81390541, 81390542, 81202266, and 81230069) and grants (2016YFC0900500, 2016YFC0900501, 2016YFC0900504, 2017YFC0907500, and 2017YFC0907504) from the National Key Research and Development Program of China. The China Kadoorie Biobank baseline survey and the first resurvey were supported by a grant from the Kadoorie Charitable Foundation in Hong Kong. The long‐term follow‐up is supported by grants from the UK Wellcome Trust (202922/Z/16/Z, 088158/Z/09/Z, 104085/Z/14/Z) and Chinese Ministry of Science and Technology (2011BAI09B01). The funders had no role in the study design, data collection, data analysis and interpretation, writing of the report, or the decision to submit the article for publication.

## Disclosures

None.

## Supporting information


**Table S1.** Characteristics of the China Kadoorie Biobank Study by Age‐Comparative Self‐Rated Health Status at Baseline
**Table S2.** Stratified Analysis: Risk of Ischemic Heart Disease According to Global Self‐Rated Health Status
**Table S3.** Sensitivity Analysis: Risk of Ischemic Heart Disease According to Age‐ Self‐Rated Health Status
**Table S4.** Stratified Analysis: Risk of Ischemic Heart Disease According to Age‐Comparative Self‐Rated Health Status
**Table S5.** Risk of Ischemic Heart Disease According to 2 Measures of Self‐Rated Health Status
**Table S6.** The Distribution of Global Self‐Rated Health Status by Age‐Comparative Self‐Rated Health Status
**Table S7.** Sensitivity Analysis: Risk of Ischemic Heart Disease According to 2 Measures of Self‐Rated Health Status
**Figure S1.** Risk of ischemic heart disease according to global self‐rated health status by study locations.
**Figure S2.** Risk of ischemic heart disease according to age‐comparative self‐rated health status by study locations.

## References

[1] Moran AE , Forouzanfar MH , Roth GA , Mensah GA , Ezzati M , Flaxman A , Murray CJ , Naghavi M . The global burden of ischemic heart disease in 1990 and 2010: the global burden of disease 2010 study. Circulation. 2014;129:1493–1501. 24573351 10.1161/CIRCULATIONAHA.113.004046 PMC4181601

[2] GBD 2013 Mortality and Causes of Death Collaborators . Global, regional, and national age‐sex specific all‐cause and cause‐specific mortality for 240 causes of death, 1990–2013: a systematic analysis for the Global Burden of Disease Study 2013. Lancet. 2015;385:117–171. 25530442 10.1016/S0140-6736(14)61682-2 PMC4340604

[3] Hu SS , Gao RL , Zhu ML , Wang W , Wang YJ , Wu ZS , Chen WW , Liu MB . Outline of the report on cardiovascular disease in china, 2010. Biomed Environ Sci. 2012;25:251–256. 22840574 10.3967/0895-3988.2012.03.001

[4] Zhou MG , Wang HD , Zhu J , Chen W , Wang L , Liu S , Li Y , Wang L , Liu Y , Yin P , Liu J , Yu S , Tan F , Barber RM , Coates MM , Dicker D , Fraser M , Gonzalez‐Medina D , Hamavid H , Hao Y , Hu G , Jiang G , Kan H , Lopez AD , Phillips MR , She J , Vos T , Wan X , Xu G , Yan LL , Yu C , Zhao Y , Zheng Y , Zou X , Naghavi M , Wang Y , Murray CJL , Yang G , Liang X . Cause‐specific mortality for 240 causes in China during 1990–2013: a systematic subnational analysis for the Global Burden of Disease Study 2013. Lancet. 2016;387:251–272. 26510778 10.1016/S0140-6736(15)00551-6

[5] Chan F , Adamo S , Coxson P , Goldman L , Gu D , Zhao D , Chen CS , He J , Mara V , Moran A . Projected impact of urbanization on cardiovascular disease in China. Int J Public Health. 2012;57:849–854. 22918518 10.1007/s00038-012-0400-y PMC3465962

[6] Appels A , Bosma H , Grabauskas V , Gostautas A , Sturmans F . Self‐rated health and mortality in a Lithuanian and a Dutch population. Soc Sci Med. 1996;42:681–689. 8685736 10.1016/0277-9536(95)00195-6

[7] Benjamins MR , Hummer RA , Eberstein IW , Nam CB . Self‐reported health and adult mortality risk: an analysis of cause‐specific mortality. Soc Sci Med. 2004;59:1297–1306. 15210100 10.1016/j.socscimed.2003.01.001

[8] Barger SD , Cribbet MR , Muldoon MF . Participant‐reported health status predicts cardiovascular and all‐cause mortality independent of established and nontraditional biomarkers: evidence from a representative US Sample. J Am Heart Assoc. 2016;5:e003741 DOI: 10.1161/JAHA.116.003741. 27572824 10.1161/JAHA.116.003741 PMC5079034

[9] Ganna A , Ingelsson E . 5 year mortality predictors in 498 103 UK Biobank participants: a prospective population‐based study. Lancet. 2015;386:533–540. 26049253 10.1016/S0140-6736(15)60175-1

[10] Fernandez‐Ruiz M , Guerra‐Vales JM , Trincado R , Fernandez R , Medrano MJ , Villarego A , Benito‐Leon J , Bermejo‐Pareja F . The ability of self‐rated health to predict mortality among community‐dwelling elderly individuals differs according to the specific cause of death: data from the NEDICES cohort. Gerontology. 2013;59:368–377. 23615509 10.1159/000348781 PMC3763238

[11] Feng Q , Zhu H , Zhen Z , Gu D . Self‐rated health, interviewer‐rated health, and their predictive powers on mortality in old age. J Gerontol B Psychol Sci Soc Sci. 2016;71:538–550. 25617400 10.1093/geronb/gbu186 PMC6366535

[12] Sargent‐Cox KA , Anstey KJ , Luszcz MA . The choice of self‐rated health measures matter when predicting mortality: evidence from 10 years follow‐up of the Australian longitudinal study of ageing. BMC Geriatr. 2010;10:18 DOI: 10.1186/1471‐2318‐10‐18. 20403203 10.1186/1471-2318-10-18 PMC2868852

[13] Kaplan GA , Goldberg DE , Everson SA , Cohen RD , Salonen R , Tuomilehto J , Salonen J . Perceived health status and morbidity and mortality: evidence from the Kuopio ischaemic heart disease risk factor study. Int J Epidemiol. 1996;25:259–265. 9119550 10.1093/ije/25.2.259

[14] Moller L , Kristensen TS , Hollnagel H . Self rated health as a predictor of incident CHD in Copenhagen, Denmark. J Epidemiol Community Health. 1996;50:423–428. 8882226 10.1136/jech.50.4.423 PMC1060313

[15] Latham K , Peek CW . Self‐rated health and morbidity onset among late midlife U.S. adults. J Gerontol B Psychol Sci Soc Sci. 2013;68:107–116. 23197340 10.1093/geronb/gbs104 PMC3605944

[16] Leung KK , Tang LY , Lue BH . Self‐rated health in Chinese institutional elderly persons. J Clin Epidemiol. 1997;50:1107–1116. 9368518 10.1016/s0895-4356(97)00153-4

[17] Shen C , Schooling CM , Chan WM , Zhou JX , Johnston JM , Lee SY , Lam TH . Self‐rated health and mortality in a prospective Chinese elderly cohort study in Hong Kong. Prev Med. 2014;67:112–118. 25045836 10.1016/j.ypmed.2014.07.018

[18] Chen Z , Lee L , Chen J , Collins R , Wu F , Guo Y , Linksted P , Peto R . Cohort profile: the Kadoorie Study of Chronic Disease in China (KSCDC). Int J Epidemiol. 2005;34:1243–1249. 16131516 10.1093/ije/dyi174

[19] Chen Z , Chen J , Collins R , Guo Y , Peto R , Wu F , Li L . China Kadoorie Biobank of 0.5 million people: survey methods, baseline characteristics and long‐term follow‐up. Int J Epidemiol. 2011;40:1652–1666. 22158673 10.1093/ije/dyr120 PMC3235021

[20] Bragg F , Li LM , Yang L , Guo Y , Chen Y , Bian Z , Chen J , Collins R , Peto R , Wang C , Dong C , Pan R , Zhou J , Xu X , Chen Z . Risks and population burden of cardiovascular diseases associated with diabetes in China: a prospective study of 0.5 million adults. PLoS Med. 2016;13:e1002026. 27379518 10.1371/journal.pmed.1002026 PMC4933372

[21] Lewington S , Lacey B , Clarke R , Guo Y , Kong XL , Yang L , Chen Y , Meng J , Xiong Y , He T , Pang Z , Zhang S , Collins R , Peto R , Li L , Chen Z . The burden of hypertension and associated risk for cardiovascular mortality in China. JAMA Intern Med. 2016;176:524–532. 26975032 10.1001/jamainternmed.2016.0190

[22] Mezuk B , Chen Y , Yu C , Guo Y , Bian Z , Collins R , Chen J , Pang Z , Wang H , Peto R , Que X , Zhang H , Tan Z , Kendler KS , Li L , Chen Z . Depression, anxiety, and prevalent diabetes in the Chinese population: findings from the China Kadoorie Biobank of 0.5 million people. J Psychosom Res. 2013;75:511–517. 24290039 10.1016/j.jpsychores.2013.09.008 PMC3919064

[23] Dowd JB , Zajacova A . Does the predictive power of self‐rated health for subsequent mortality risk vary by socioeconomic status in the US? Int J Epidemiol. 2007;36:1214–1221. 17971388 10.1093/ije/dym214

[24] Lima‐Costa MF , Steptoe A , Cesar CC , De Oliveira C , Proietti FA , Marmot M . The influence of socioeconomic status on the predictive power of self‐rated health for 6‐year mortality in English and Brazilian older adults: the ELSA and Bambui cohort studies. Ann Epidemiol. 2012;22:644–648. 22819435 10.1016/j.annepidem.2012.06.101

[25] Dowd JB , Zajacova A . Does self‐rated health mean the same thing across socioeconomic groups? Evidence from biomarker data Ann Epidemiol. 2010;20:743–749. 20816313 10.1016/j.annepidem.2010.06.007 PMC4845753

[26] Yu C , Shi Z , Lv J , Du H , Qi L , Guo Y , Bian Z , Chang L , Tang X , Jiang Q , Mu H , Pan D , Chen J , Chen Z , Li L . Major dietary patterns in relation to general and central obesity among Chinese adults. Nutrients. 2015;7:5834–5849. 26184308 10.3390/nu7075253 PMC4517030

[27] Millwood IY , Li L , Smith M , Guo Y , Yang L , Bian Z , Lewington S , Whitlock G , Sherliker P , Collins R , Chen J , Peto R , Wang H , Xu J , He J , Yu M , Liu H , Chen Z . Alcohol consumption in 0.5 million people from 10 diverse regions of China: prevalence, patterns and socio‐demographic and health‐related correlates. Int J Epidemiol. 2013;42:816–827. 23918852 10.1093/ije/dyt078 PMC3733702

[28] Zarini GG , Vaccaro JA , Canossa Terris MA , Exebio JC , Tokayer L , Antwi J , Ajabshir S , Cheema A , Huffman FG . Lifestyle behaviors and self‐rated health: the living for health program. J Environ Public Health. 2014;2014:315042. 25530764 10.1155/2014/315042 PMC4228703

[29] Eriksson I , Unden AL , Elofsson S . Self‐rated health. Comparisons between three different measures. Results from a population study. Int J Epidemiol. 2001;30:326–333. 11369738 10.1093/ije/30.2.326

[30] Mood C . Life‐style and self‐rated global health in Sweden: a prospective analysis spanning three decades. Prev Med. 2013;57:802–806. 24041976 10.1016/j.ypmed.2013.09.002

[31] Chen Y , While AE , Hicks A . Self‐rated health and associated factors among older people living alone in Shanghai. Geriatr Gerontol Int. 2015;15:457–464. 24750391 10.1111/ggi.12298

[32] Stanojevic Jerkovic O , Sauliune S , Sumskas L , Birt C , Kersnik J . Determinants of self‐rated health in elderly populations in urban areas in Slovenia, Lithuania and UK: findings of the EURO‐URHIS 2 survey. Eur J Public Health. 2017;27(suppl 2):74–79. 26163468 10.1093/eurpub/ckv097

[33] Waller G , Janlert U , Hamberg K , Forssen A . What does age‐comparative self‐rated health measure? A cross‐sectional study from the Northern Sweden MONICA Project. Scand J Public Health. 2016;44:233–239. 26644159 10.1177/1403494815618554

[34] Heidrich J , Liese AD , Lowel H , Keil U . Self‐rated health and its relation to all‐cause and cardiovascular mortality in southern Germany. results from the MONICA Augsburg Cohort Study 1984–1995. Ann Epidemiol. 2002;12:338–345. 12062922 10.1016/s1047-2797(01)00300-3

[35] Baron‐Epel O , Shemy G , Carmel S . Prediction of survival: a comparison between two subjective health measures in an elderly population. Soc Sci Med. 2004;58:2035–2043. 15020018 10.1016/S0277-9536(03)00412-X

[36] Manderbacka K , Kareholt I , Martikainen P , Lundberg O . The effect of point of reference on the association between self‐rated health and mortality. Soc Sci Med. 2003;56:1447–1452. 12614696 10.1016/s0277-9536(02)00141-7

[37] Vuorisalmi M , Lintonen T , Jylha M . Global self‐rated health data from a longitudinal study predicted mortality better than comparative self‐rated health in old age. J Clin Epidemiol. 2005;58:680–687. 15939219 10.1016/j.jclinepi.2004.11.025

[38] Waller G , Thalen P , Janlert U , Hamberg K , Forssen A . A cross‐sectional and semantic investigation of self‐rated health in the northern Sweden MONICA‐study. BMC Med Res Methodol. 2012;12:154. 23046741 10.1186/1471-2288-12-154 PMC3537697

[39] Haseli‐Mashhadi N , Pan A , Ye X , Wang J , Qi Q , Liu Y , Li H , Yu Z , Lin X , Franco OH . Self‐rated health in middle‐aged and elderly Chinese: distribution, determinants and associations with cardio‐metabolic risk factors. BMC Public Health. 2009;9:368. 19788754 10.1186/1471-2458-9-368 PMC2760533

[40] Brenowitz WD , Hubbard RA , Crane PK , Gray SL , Zaslavsky O , Larson EB . Longitudinal associations between self‐rated health and performance‐based physical function in a population‐based cohort of older adults. PLoS One. 2014;9:e111761. 25365288 10.1371/journal.pone.0111761 PMC4218810

[41] Shanahan L , Bauldry S , Freeman J , Bondy CL . Self‐rated health and C‐reactive protein in young adults. Brain Behav Immun. 2014;36:139–146. 24513874 10.1016/j.bbi.2013.10.020 PMC4313081

[42] Tanno K , Ohsawa M , Onoda T , Itai K , Sakata K , Tanaka F , Makita S , Nakamura M , Omama S , Ogasawara M , Ogawa A , Ishibashi Y , Kuribayashi T , Koyama T , Okayama A . Poor self‐rated health is significantly associated with elevated C‐reactive protein levels in women, but not in men, in the Japanese general population. J Psychosom Res. 2012;73:225–231. 22850264 10.1016/j.jpsychores.2012.05.013

[43] Christian LM , Glaser R , Porter K , Malarkey WB , Beversdorf D , Kiecolt‐Glaser JK . Poorer self‐rated health is associated with elevated inflammatory markers among older adults. Psychoneuroendocrinology. 2011;36:1495–1504. 21601365 10.1016/j.psyneuen.2011.04.003 PMC3161147

[44] Jarczok MN , Kleber ME , Koenig J , Loerbroks A , Herr RM , Hoffmann K , Fischer JE , Benyamini Y , Thayer JF . Investigating the associations of self‐rated health: heart rate variability is more strongly associated than inflammatory and other frequently used biomarkers in a cross sectional occupational sample. PLoS One. 2015;10:e0117196. 25693164 10.1371/journal.pone.0117196 PMC4333766

[45] Jylha M . What is self‐rated health and why does it predict mortality? Towards a unified conceptual model. Soc Sci Med. 2009;69:307–316. 19520474 10.1016/j.socscimed.2009.05.013

