# Supplementary material for: Self‐Rated Health Status and Risk of Ischemic Heart Disease in the China Kadoorie Biobank Study: A Population‐Based Cohort Study
Source: J Am Heart Assoc. 2017 Sep 22;6(9):e006595. doi: 10.1161/JAHA.117.006595 (PMC5634301; doi:10.1161/JAHA.117.006595)
Supplement: Supplementary file 1 — Table S1. Characteristics of the China Kadoorie Biobank Study by Age‐Comparative Self‐Rated Health Status at Baseline Table S2. Stratified Analysis: Risk of Ischemic Heart Disease According to Global Self‐Rated Health Status Table S3. Sensitivity Analysis: Risk of Ischemic Heart Disease According to Age‐ Self‐Rated Health Status Table S4. Stratified Analysis: Risk of Ischemic Heart Disease According to Age‐Comparative Self‐Rated Health Status Table S5. Risk of Ischemic Heart Disease According to 2 Measures of Self‐Rated Health Status Table S6. The Distribution of Global Self‐Rated Health Status by Age‐Comparative Self‐Rated Health Status Table S7. Sensitivity Analysis: Risk of Ischemic Heart Disease According to 2 Measures of Self‐Rated Health Status Figure S1. Risk of ischemic heart disease according to global self‐rated health status by study locations. Figure S2. Risk of ischemic heart disease according to age‐comparative self‐rated health status by study locations. [file JAH3-6-e006595-s001.pdf]

SUPPLEMENTAL MATERIAL

Table S1. Characteristics of the China Kadoorie Biobank Study by Age-comparative Self-rated Health Status at Baseline

Variables	Total, N (%)	Age-comparative self-rated health, N (%)			
		Better	Same	Worse	Don't know
**N**	486541 (100)	90738 (18.7)	309022 (63.5)	71791 (14.8)	14990 (3.1)
**Age, years**†	51.0 (10.5)	50.8 (10.7)	50.9 (10.5)	51.4 (10.5)	53.8 (11.0)
**Sex**					
**Male**	199113 (40.9)	41752 (46.0)	127283 (41.2)	24690 (34.4)	5388 (35.9)
**Female**	287428 (59.1)	48986 (54.0)	181739 (58.8)	47101 (65.6)	9602 (64.1)
**Marital status**					
**Married**	44228 (90.9)	82626 (91.0)	282654 (91.4)	63894 (89.0)	13144 (87.7)
**Widowed**	33081 (6.8)	5853 (6.5)	20015 (6.5)	5793 (8.1)	1420 (9.5)
**Separated/divorced**	7579 (1.6)	1765 (2.0)	4178 (1.4)	1372 (1.9)	264 (1.7)
**Never married**	3653 (0.7)	494 (0.5)	2265 (0.7)	732 (1.0)	162 (1.1)
**Education level**					
**No formal school**	90829 (18.7)	12931 (14.3)	57403 (18.6)	17331 (24.1)	3164 (21.1)
**Primary**	156407 (32.1)	24060 (26.5)	104137 (33.7)	23403 (32.6)	4807 (32.1)
**Middle or high school**	211683 (43.5)	45443 (50.1)	131789 (42.6)	28146 (39.2)	6305 (42.0)
**College/university or more**	27622 (5.7)	8304 (9.1)	15693 (5.1)	2911 (4.1)	714 (4.8)
**Household income,**					

**RMB**					
**<10,000**	137893 (28.3)	24621 (27.1)	80679 (26.1)	28885 (40.2)	3708 (24.7)
**10,000-19,999**	140432 (28.9)	26004 (28.7)	90375 (29.3)	20027 (27.9)	4026 (26.9)
**20,000-34,999**	120197 (24.7)	21718 (23.9)	81437 (26.3)	13752 (19.2)	3290 (21.9)
**≥35,000**	88019 (18.1)	18395 (20.3)	56531 (18.3)	9127 (12.7)	3966 (26.5)
**Occupation**					
**Farmers**	208754 (42.9)	33863 (37.3)	137213 (44.4)	33154 (46.2)	4524 (30.2)
**Factory workers**	71331 (14.7)	14548 (16.0)	46761 (15.1)	8466 (11.8)	1556 (10.4)
**Professionals and managers**	64533 (13.3)	16501 (18.2)	38609 (12.5)	7414 (10.3)	2009 (13.4)
**Retired**	72156 (14.8)	16124 (17.8)	43202 (14.0)	9286 (12.9)	3554 (23.6)
**Unemployed and others**	69767 (14.3)	9702 (10.7)	43237 (14.0)	13471 (18.8)	3357 (22.4)
**Administrative Region**					
**Rural**	276755 (56.9)	41227 (45.4)	188614 (61.0)	42215 (58.8)	4699 (31.3)
**Urban**	209786 (43.1)	49511 (54.6)	120408 (39.0)	29576 (41.2)	10291 (68.7)
**Geographical region**					
**North**	190992 (39.3)	45072 (49.7)	115313 (37.3)	29285 (40.8)	1322 (8.8)
**South**	295549 (60.7)	45666 (50.3)	193709 (62.7)	42506 (59.2)	13668 (91.2)
**Sleep problems (Yes)**	79756 （16.4）	10244 (11.3)	45539 (14.7)	21159 (29.5)	2814 (18.8)
**Cigarette smoking**					
**Never**	301601 (62.0)	52025 (57.3)	192713 (62.4)	46801 (65.2)	10062 (67.1)

**Former**	26807 (5.5)	5428 (6.0)	16253 (5.2)	4385 (6.1)	741 (5.0)
**Occasionally**	27872 (5.7)	5878 (6.5)	17153 (5.6)	3995 (5.6)	846 (5.6)
**Current**	130261 (26.8)	27407 (30.2)	82903 (26.8)	16610 (23.1)	3341 (22.3)
**Alcohol drinking**					
**Never**	222161 (45.7)	31476 (34.7)	145507 (47.1)	36254 (50.5)	8924 (59.5)
**Ex-drinker**	7751 (1.6)	954 (1.1)	4419 (1.4)	2118 (3.0)	260 (1.7)
**Occasionally**	172550 (35.4)	37489 (41.3)	107436 (34.8)	23796 (33.1)	3829 (25.6)
**Weekly**	84079 (17.3)	80819 (22.9)	51660 (16.7)	9623 (13.4)	1977 (13.2)
**Physical activity, MET-hr/wk‡**	18.1 (10.8, 30.6)	18.8 (11.2, 30.2)	18.4 (11.0, 31.4)	11.9 (9.6, 28.8)	14.3 (8.4, 24.2)
**Menopausal status (women only)**					
**Premenopause**	127325 (44.3)	23446 (47.9)	81960 (45.1)	18548 (39.4)	3371 (35.1)
**Perimenopause**	14354 (5.0)	2484 (5.1)	9157 (5.0)	2334 (4.9)	379 (4.0)
**Postmenopause**	145749 (50.7)	23056 (47.0)	90622 (49.9)	26219 (55.7)	5852 (60.9)
**BMI, kg/m^2**					
**<18.5**	21428 (4.4)	2244 (2.5)	13167 (4.2)	5117 (7.1)	900 (6.0)
**18.5-23.9**	255773 (52.6)	44565 (49.1)	165503 (53.6)	37892 (52.8)	7813 (52.1)
**24.0-27.9**	159677 (32.8)	33593 (37.0)	100406 (32.5)	20847 (29.0)	4831 (32.2)
**≥28.0**	49663 (10.2)	10336 (11.4)	29946 (9.7)	7935 (11.1)	1446 (9.7)
**Family history of heart disease (Yes)**	15379 (3.2)	3399 (3.8)	8946 (2.9)	2663 (3.7)	371 (2.5)

**Baseline major depression (Yes)**	2972 (0.6)	309 (0.3)	1308 (0.4)	1274 (1.8)	81 (0.5)
**Baseline diabetes (Yes)**	26118 (5.4)	3380 (3.7)	14855 (4.8)	6878 (9.6)	1005 (6.7)
**Baseline hypertension (Yes)**	158473 (32.6)	26673 (29.4)	101085 (32.7)	25526 (35.6)	5189 (34.6)
**Other baseline comorbidities (Yes)**	229350 (47.1)	38134 (42.0)	141452 (45.8)	42224 (58.8)	7540 (50.3)

* Two-sided P values were derived from ANOVA for continuous variables and Chi-square test for categorical variables, all *P* values are <0.001.

† Data are shown as mean (standard deviation).

‡ Data are shown as median (P25, P75).

Table S2. Stratified Analysis: Risk of Ischemic Heart Disease According to Global Self-rated Health Status

**Subgroups**	**Cases/person-years**	**Model 1*** **HR (95% CI)**	**Model 2†** **HR (95% CI)**	**Model 3‡** **HR (95% CI)**	***P* for interaction**
**Age**					0.10
**30-59 years old**					
**Excellent**	2070/515951	1.00	1.00	1.00	
**Good**	2950/818435	1.05 (0.99, 1.11)	1.04 (0.99, 1.11)	1.03 (1.97, 1.09)	
**Fair**	6164/1133304	1.40 (1.33, 1.48)	1.39 (1.32, 1.46)	1.33 (1.27, 1.40)	
**Poor**	1712/234020	2.02 (1.90, 2.16)	1.96 (1.84, 2.10)	1.80 (1.68, 1.92)	
**60-69 years old**					
**Excellent**	1091/82991	1.00	1.00	1.00	
**Good**	1650/148674	1.08 (1.00, 1.17)	1.08 (0.99, 1.16)	1.06 (0.98, 1.15)	
**Fair**	3998/256820	1.44 (1.34, 1.54)	1.41 (1.32, 1.51)	1.37 (1.27, 1.47)	
**Poor**	1112/57703	1.95 (1.79, 2.13)	1.87 (1.72, 2.05)	1.75 (1.60, 1.91)	
**70-79 years old**					
**Excellent**	577/25403	1.00	1.00	1.00	
**Good**	805/45009	0.96 (0.86, 1.07)	0.95 (0.85, 1.06)	0.94 (0.84, 1.05)	
**Fair**	1986/85692	1.24 (1.13, 1.37)	1.22 (1.10, 1.34)	1.19 (1.08, 1.31)	
**Poor**	590/19539	1.82 (1.62, 2.06)	1.74 (1.54, 1.96)	1.66 (1.47, 1.88)	
**Sex**					0.59
**Men**					

**Excellent**	1760/287799	1.00	1.00	1.00	
**Good**	2459/426251	1.06 (1.00, 1.13)	1.06 (1.00, 1.13)	1.05 (0.99, 1.12)	
**Fair**	4830/568999	1.39 (1.31, 1.47)	1.37 (1.30, 1.45)	1.32 (1.25, 1.40)	
**Poor**	1133/103122	1.96 (1.81, 2.12)	1.91 (1.76, 2.06)	1.76 (1.62, 1.90)	
**Women**					
**Excellent**	1978/336547	1.00	1.00	1.00	
**Good**	2946/585867	1.03 (0.97, 1.09)	1.02 (0.96, 1.08)	1.00 (0.95, 1.06)	
**Fair**	7318/906817	1.38 (1.31, 1.45)	1.36 (1.29, 1.43)	1.31 (1.25, 1.38)	
**Poor**	2281/208140	1.96 (1.84, 2.09)	1.89 (1.77, 2.01)	1.76 (1.65, 1.87)	
**Administrative region**					0.43
**Rural**					
**Excellent**	1407/276017	1.00	1.00	1.00	
**Good**	3228/650831	1.03 (0.96, 1.09)	1.02 (0.96, 1.09)	1.01 (0.95, 1.08)	
**Fair**	6331/847499	1.33 (1.25, 1.41)	1.31 (1.23, 1.39)	1.28 (1.21, 1.36)	
**Poor**	2096/202076	1.90 (1.77, 2.03)	1.83 (1.71, 1.96)	1.73 (1.61, 1.86)	
**Urban**					
**Excellent**	2331/348328	1.00	1.00	1.00	
**Good**	2177/361287	1.05 (0.99, 1.11)	1.04 (0.98, 1.11)	1.03 (0.97, 1.09)	
**Fair**	5817/628317	1.43 (1.36, 1.51)	1.41 (1.34, 1.48)	1.35 (1.28, 1.42)	
**Poor**	1318/109186	2.00 (1.87, 2.15)	1.92 (1.79, 2.06)	1.74 (1.63, 1.87)	
**Geographical**					<0.001

**region**					
**North**					
**Excellent**	2795/297781	1.00	1.00	1.00	
**Good**	3149/377323	1.04 (0.99, 1.10)	1.04 (0.99, 1.10)	1.03 (0.97, 1.08)	
**Fair**	6169/541521	1.35 (1.29, 1.41)	1.33 (1.27, 1.40)	1.29 (1.23, 1.35)	
**Poor**	1888/118204	1.86 (1.76, 1.98)	1.80 (1.70s, 1.92)	1.67 (1.57, 1.78)	
**South**					
**Excellent**	943/326565	1.00	1.00	1.00	
**Good**	2256/634795	1.07 (0.99, 1.16)	1.06 (0.99, 1.15)	1.05 (0.98, 1.14)	
**Fair**	5979/934295	1.47 (1.37, 1.58)	1.45 (1.36, 1.56)	1.41 (1.31, 1.51)	
**Poor**	1526/193059	2.16 (1.99, 2.35)	2.09 (1.93, 2.28)	1.95 (1.79, 2.13)	
**Education**					0.01
**No formal education**					
**Excellent**	642/94398	1.00	1.00	1.00	
**Good**	1379/212064	1.02 (0.93, 1.12)	1.00 (0.91, 1.11)	1.00 (0.90, 1.09)	
**Fair**	2800/258668	1.34 (1.22, 1.46)	1.30 (1.19, 1.42)	1.27 (1.16, 1.39)	
**Poor**	1010/76573	1.75 (1.58, 1.94)	1.66 (1.50, 1.84)	1.59 (1.44, 1.77)	
**Primary school**					
**Excellent**	958/163372	1.00	1.00	1.00	
**Good**	1776/327991	1.03 (0.95, 1.12)	1.03 (0.96, 1.12)	1.02 (0.95, 1.11)	
**Fair**	4169/495850	1.33 (1.24, 1.43)	1.32 (1.23, 1.42)	1.28 (1.19, 1.38)	

**Poor**	1205/110663	2.03 (1.86, 2.21)	1.98 (1.81, 2.16)	1.85 (1.69, 2.02)	
**Middle school or higher**					
**Excellent**	2138/366575	1.00	1.00	1.00	
**Good**	2250/472063	1.05 (0.98, 1.11)	1.04 (0.98, 1.10)	1.03 (0.97, 1.09)	
**Fair**	5179/721298	1.43 (1.36, 1.51)	1.41 (1.34, 1.48)	1.35 (1.28, 1.42)	
**Poor**	1199/124027	2.02 (1.88, 2.17)	1.94 (1.80, 2.09)	1.76 (1.63, 1.90)	
**Income**					0.03
**<10,000**					
**Excellent**	963/138097	1.00	1.00	1.00	
**Good**	1534/268338	0.95 (0.88, 1.04)	0.95 (0.88, 1.04)	0.95 (0.87, 1.03)	
**Fair**	3569/442055	1.24 (1.15, 1.33)	1.22 (1.14, 1.32)	1.20 (1.11, 1.29)	
**Poor**	1563/142699	1.79 (1.65, 1.94)	1.73 (1.59, 1.88)	1.63 (1.50, 1.77)	
**10,000-34,999**					
**Excellent**	2019/339702	1.00	1.00	1.00	
**Good**	2833/543642	1.06 (1.00, 1.13)	1.05 (0.99, 1.12)	1.04 (0.98, 1.10)	
**Fair**	6721/811926	1.41 (1.34, 1.49)	1.39 (1.32, 1.47)	1.34 (1.27, 1.41)	
**Poor**	1555/138517	2.01 (1.88, 2.16)	1.94 (1.81, 2.08)	1.79 (1.67, 1.92)	
**≥35,000**					
**Excellent**	756/146546	1.00	1.00	1.00	
**Good**	1038/200138	1.11 (1.01, 1.22)	1.10 (1.00, 1.21)	1.09 (0.99, 1.20)	
**Fair**	1858/221835	1.52 (1.39, 1.66)	1.49 (1.36, 1.63)	1.44 (1.32, 1.57)	

**Poor**	296/30047	2.01 (1.75, 2.30)	1.92 (1.67, 2.21)	1.78 (1.55, 2.05)	
**Cigarette smoking**					0.65
**Never**					
**Excellent**	2102/364220	1.00	1.00	1.00	
**Good**	3092/618264	1.03 (0.97, 1.09)	1.02 (0.97, 1.08)	1.01 (0.95, 1.07)	
**Fair**	7544/949098	1.39 (1.32, 1.46)	1.37 (1.31, 1.44)	1.33 (1.26, 1.39)	
**Poor**	2126/203022	1.94 (1.82, 2.06)	1.87 (1.76, 2.00)	1.74 (1.64, 1.86)	
**Former**					
**Excellent**	316/33514	1.00	1.00	1.00	
**Good**	460/53773	1.09 (0.94, 1.27)	1.09 (0.94, 1.26)	1.08 (0.93, 1.25)	
**Fair**	991/76382	1.46 (1.28, 1.67)	1.42 (1.25, 1.63)	1.38 (1.21, 1.58)	
**Poor**	312/18895	2.07 (1.76, 2.45)	2.00 (1.69, 2.37)	1.90 (1.60, 2.25)	
**Current**					
**Excellent**	1320/226611	1.00	1.00	1.00	
**Good**	1853/340081	1.05 (0.98, 1.13)	1.05 (0.98, 1.13)	1.04 (0.96, 1.11)	
**Fair**	3613/450336	1.34 (1.26, 1.43)	1.33 (1.24, 1.42)	1.28 (1.20, 1.36)	
**Poor**	976/89345	1.95 (1.79, 2.13)	1.90 (1.75, 2.08)	1.75 (1.60, 1.91)	
**Alcohol drinking**					0.14
**Never**					
**Excellent**	1467/249874	1.00	1.00	1.00	
**Good**	2433/458946	1.00 (0.94, 1.07)	1.00 (0.93, 1.06)	0.98 (0.92, 1.05)	

**Fair**	6171/701208	1.36 (1.28, 1.44)	1.35 (1.27, 1.43)	1.30 (1.22, 1.38)	
**Poor**	1814/156203	1.81 (1.69, 1.94)	1.78 (1.65, 1.91)	1.65 (1.53, 1.77)	
**Former**					
**Excellent**	56/5892	1.00	1.00	1.00	
**Good**	119/11501	1.18 (0.85, 1.64)	1.19 (0.86, 1.65)	1.18 (0.85, 1.63)	
**Fair**	317/24739	1.30 (0.97, 1.74)	1.30 (0.97, 1.74)	1.28 (0.95, 1.72)	
**Poor**	148/9801	1.93 (1.40, 2.66)	1.92 (1.39, 2.66)	1.88 (1.36, 2.61)	
**Current**					
**Excellent**	2215/368579	1.00	1.00	1.00	
**Good**	2853/541671	1.07 (1.01, 1.13)	1.07 (1.01, 1.13)	1.05 (1.00, 1.12)	
**Fair**	5660/749869	1.39 (1.32, 1.46)	1.38 (1.31, 1.45)	1.33 (1.27, 1.40)	
**Poor**	1452/145258	2.06 (1.93, 2.21)	2.03 (1.89, 2.17)	1.88 (1.75, 2.01)	
**Physical activity**					0.19
**Low**					
**Excellent**	1695/160605	1.00	1.00	1.00	
**Good**	2303/267400	1.04 (0.98, 1.11)	1.04 (0.97, 1.10)	1.02 (0.96, 1.09)	
**Fair**	6253/505584	1.40 (1.32, 1.48)	1.38 (1.30, 1.46)	1.33 (1.25, 1.40)	
**Poor**	1959/117306	2.06 (1.92, 2.20)	1.99 (1.86, 2.13)	1.83 (1.71, 1.96)	
**Moderate**					
**Excellent**	1279/230251	1.00	1.00	1.00	
**Good**	1664/336986	1.03 (0.95, 1.11)	1.02 (0.94, 1.09)	1.00 (0.93, 1.08)	
**Fair**	3498/495711	1.35 (1.26, 1.44)	1.32 (1.24, 1.41)	1.27 (1.19, 1.36)	

**Poor**	875/103363	1.82 (1.66, 1.99)	1.75 (1.60, 1.92)	1.62 (1.48, 1.78)	
**High**					
**Excellent**	764/233490	1.00	1.00	1.00	
**Good**	1438/407732	1.07 (0.97, 1.16)	1.06 (0.97, 1.16)	1.05 (0.96, 1.15)	
**Fair**	2397/474521	1.40 (1.29, 1.52)	1.38 (1.27, 1.50)	1.35 (1.24, 1.47)	
**Poor**	580/90593	1.86 (1.66, 2.08)	1.81 (1.61, 2.02)	1.73 (1.54, 1.94)	
**BMI**					0.32
**<18.5 kg/m^2**					
**Excellent**	106/17870	1.00	1.00	1.00	
**Good**	212/33597	1.12 (0.88, 1.42)	1.11 (0.88, 1.41)	1.10 (0.87, 1.40)	
**Fair**	657/71552	1.51 (1.22, 1.87)	1.48 (1.19, 1.83)	1.44 (1.17, 1.79)	
**Poor**	292/22222	2.18 (1.73, 2.75)	2.06 (1.63, 2.60)	1.98 (1.57, 2.52)	
**18.5-23.9 kg/m^2**					
**Excellent**	1580/322407	1.00	1.00	1.00	
**Good**	2455/535525	1.05 (0.98, 1.12)	1.04 (0.98, 1.11)	1.03 (0.97, 1.10)	
**Fair**	5445/785795	1.38 (1.30, 1.46)	1.36 (1.29, 1.45)	1.32 (1.25, 1.40)	
**Poor**	1501/163667	1.98 (1.84, 2.13)	1.92 (1.79, 2.07)	1.79 (1.66, 1.93)	
**24.0-27.9 kg/m^2**					
**Excellent**	1490/220322	1.00	1.00	1.00	
**Good**	1929/342523	1.01 (0.94, 1.08)	1.01 (0.94, 1.08)	0.99 (0.93, 1.06)	
**Fair**	4249/470164	1.37 (1.29, 1.46)	1.35 (1.27, 1.44)	1.30 (1.22, 1.38)	
**Poor**	1069/91315	1.89 (1.74, 2.05)	1.81 (1.67, 1.97)	1.67 (1.54, 1.81)	

**≥28.0 kg/m²**					
**Excellent**	562/63747	1.00	1.00	1.00	
**Good**	809/100473	1.10 (0.99, 1.23)	1.09 (0.98, 1.22)	1.08 (0.97, 1.21)	
**Fair**	1797/148305	1.40 (1.27, 1.55)	1.38 (1.25, 1.52)	1.33 (1.20, 1.46)	
**Poor**	552/34058	1.96 (1.73, 2.21)	1.89 (1.67, 2.14)	1.74 (1.53, 1.97)	
**History of diabetes**					0.78
**No**					
**Excellent**	3431/603383	1.00	1.00	1.00	
**Good**	4964/975069	1.04 (0.99, 1.09)	1.03 (0.99, 1.08)	1.02 (0.98, 1.07)	
**Fair**	10741/1391903	1.37 (1.32, 1.43)	1.36 (1.30, 1.41)	1.33 (1.27, 1.38)	
**Poor**	2807/281472	1.92 (1.82, 2.02)	1.87 (1.77, 1.97)	1.78 (1.69, 1.88)	
**Yes**					
**Excellent**	307/20962	1.00	1.00	1.00	
**Good**	441/37050	1.03 (0.89, 1.20)	1.03 (0.89, 1.19)	1.03 (0.89, 1.19)	
**Fair**	1407/83913	1.29 (1.14, 1.47)	1.27 (1.12, 1.44)	1.25 (1.10, 1.42)	
**Poor**	607/29790	1.69 (1.47, 1.95)	1.62 (1.40, 1.87)	1.57 (1.36, 1.81)	
**History of hypertension**					0.13
**No**					
**Excellent**	2057/451148	1.00	1.00	1.00	
**Good**	2747/694975	1.05 (0.99, 1.11)	1.05 (0.99, 1.11)	1.04 (0.98, 1.10)	

**Fair**	5625/987276	1.34 (1.27, 1.41)	1.33 (1.26, 1.40)	1.30 (1.24, 1.37)
**Poor**	1499/199089	1.93 (1.80, 2.06)	1.89 (1.76, 2.03)	1.80 (1.68, 1.93)
**Yes**				
**Excellent**	1681/173198	1.00	1.00	1.00
**Good**	2658/317143	1.02 (0.96, 1.09)	1.01 (0.95, 1.08)	1.01 (0.95, 1.08)
**Fair**	6523/488539	1.37 (1.30, 1.45)	1.35 (1.28, 1.43)	1.33 (1.26, 1.41)
**Poor**	1915/112174	1.87 (1.75, 2.00)	1.80 (1.68, 1.92)	1.73 (1.62, 1.86)

[*] Model 1: adjusted for age, marital status, education level, household income, occupation, and menopausal status;

[†] Model 2: model 1 plus sleep problems, cigarette smoking, alcohol drinking, physical activity, body mass index, and family history of heart disease;

[‡] Model 3: model 2 plus baseline major depression, diabetes, hypertension and other comorbidities.

Table S3. Sensitivity Analysis: Risk of Ischemic Heart Disease According to Age- Self-rated Health Status

Age-comparative self-rated health	Cases/person-years	Model 1* HR (95% CI)	Model 2† HR (95% CI)	Model 3‡ HR (95% CI)
**Including "don't know"**				
Better	4038/649835	1.00	1.00	1.00
Same	14939/2172181	1.27 (1.22, 1.31)	1.26 (1.21, 1.31)	1.23 (1.19, 1.27)
Worse	5048/501051	1.96 (1.87, 2.04)	1.91 (1.83, 1.99)	1.78 (1.70, 1.86)
don't know	680/100476	1.53 (1.41,1.67)	1.51 (1.39,1.64)	1.46 (1.34,1.59)

* Model 1: adjusted for age, marital status, education level, household income, occupation, and menopausal status;

† Model 2: model 1 plus sleep problems, cigarette smoking, alcohol drinking, physical activity, body mass index, and family history of heart disease;

‡ Model 3: model 2 plus baseline major depression, diabetes, hypertension and other comorbidities.

Table S4. Stratified Analysis: Risk of Ischemic Heart Disease According to Age-comparative Self-rated Health Status

Subgroups	Cases/person-years	Model 1* HR (95% CI)	Model 2† HR (95% CI)	Model 3‡ HR (95% CI)	*P* for interaction
**Age**					0.14
**30-59 years old**					
**Better**	2015/511321	1.00	1.00	1.00	
**Same**	7828/1726348	1.26 (1.20, 1.32)	1.26 (1.20, 1.32)	1.23 (1.17, 1.29)	
**Worse**	2761/394268	1.97 (1.86, 2.09)	1.93 (1.82, 2.05)	1.78 (1.68, 1.89)	
**60-69 years old**					
**Better**	1311/103743	1.00	1.00	1.00	
**Same**	4769/339072	1.30 (1.22, 1.39)	1.29 (1.21, 1.37)	1.26 (1.18, 1.34)	
**Worse**	1548/82252	1.94 (1.80, 2.09)	1.88 (1.74, 2.03)	1.76 (1.62, 1.90)	
**70-79 years old**					
**Better**	712/34771	1.00	1.00	1.00	
**Same**	2342/106761	1.24 (1.13, 1.35)	1.22 (1.12, 1.33)	1.20 (1.10, 1.31)	
**Worse**	739/24531	1.97 (1.77, 2.19)	1.89 (1.69, 2.11)	1.82 (1.63, 2.03)	
**Sex**					0.34
**Men**					
**Better**	1955/296829	1.00	1.00	1.00	
**Same**	6243/885507	1.22 (1.16, 1.29)	1.22 (1.16, 1.29)	1.19 (1.13, 1.25)	
**Worse**	1720/168252	1.93 (1.80, 2.06)	1.90 (1.78, 2.03)	1.77 (1.65, 1.89)	

**Women**					
**Better**	2083/353006	1.00	1.00	1.00	
**Same**	8696/1286673	1.31 (1.24, 1.37)	1.29 (1.23, 1.36)	1.26 (1.20, 1.33)	
**Worse**	3328/332799	1.99 (1.88, 2.10)	1.92 (1.82, 2.04)	1.80 (1.70, 1.90)	
**Administrative region**					0.96
**Rural**					
**Better**	1484/301125	1.00	1.00	1.00	
**Same**	8453/1344074	1.25 (1.18, 1.32)	1.24 (1.17, 1.31)	1.22 (1.15, 1.29)	
**Worse**	2855/297499	1.88 (1.76, 2.01)	1.83 (1.71, 1.95)	1.74 (1.63, 1.86)	
**Urban**					
**Better**	2554/348710	1.00	1.00	1.00	
**Same**	6486/828107	1.28 (1.22, 1.34)	1.27 (1.21, 1.33)	1.23 (1.18, 1.29)	
**Worse**	2193/203552	2.02 (1.90, 2.14)	1.95 (1.84, 2.07)	1.79 (1.68, 1.90)	
**Geographical region**					<0.001
**North**					
**Better**	3047/321838	1.00	1.00	1.00	
**Same**	7895/799026	1.26 (1.21, 1.32)	1.26 (1.21, 1.31)	1.23 (1.18, 1.28)	
**Worse**	2917/204427	1.85 (1.76, 1.95)	1.81 (1.71, 1.90)	1.68 (1.59, 1.77)	
**South**					
**Better**	991/327997	1.00	1.00	1.00	

**Same**	7044/1373155	1.29 (1.21, 1.39)	1.29 (1.20, 1.38)	1.26 (1.18, 1.35)	
**Worse**	2131/296624	2.15 (1.99, 2.32)	2.10 (1.94, 2.27)	1.98 (1.83, 2.14)	
**Education**					0.09
**No formal school**					
**Better**	663/93968	1.00	1.00	1.00	
**Same**	3515/406313	1.25 (1.15, 1.36)	1.23 (1.13, 1.34)	1.21 (1.11, 1.32)	
**Worse**	1469/120336	1.78 (1.62, 1.95)	1.70 (1.55, 1.87)	1.63 (1.48, 1.80)	
**Primary**					
**Better**	1030/172323	1.00	1.00	1.00	
**Same**	5260/730695	1.24 (1.15, 1.33)	1.23 (1.15, 1.32)	1.20 (1.12, 1.29)	
**Worse**	1611/161926	1.98 (1.83, 2.15)	1.94 (1.79, 2.1,)	1.81 (1.67, 1.97)	
**Middle school or higher**					
**Better**	2345/383544	1.00	1.00	1.00	
**Same**	6164/1035173	1.28 (1.22, 1.35)	1.28 (1.22, 1.34)	1.24 (1.18, 1.30)	
**Worse**	1968/218789	2.04 (1.92, 2.17)	1.99 (1.87, 2.12)	1.83 (1.72, 1.95)	
**Income**					0.04
**<10,000**					
**Better**	1160/179879	1.00	1.00	1.00	
**Same**	4233/581341	1.17 (1.09, 1.25)	1.16 (1.09, 1.24)	1.14 (1.07, 1.22)	
**Worse**	2071/203782	1.74 (1.62, 1.88)	1.69 (1.57, 1.82)	1.60 (1.48, 1.73)	
**10,000-34,999**					

**Better**	2013/342640	1.00	1.00	1.00	
**Same**	8244/1205969	1.31 (1.25, 1.38)	1.30 (1.24, 1.37)	1.27 (1.21, 1.33)	
**Worse**	2458/235471	2.09 (1.97, 2.21)	2.03 (1.91, 2.16)	1.89 (1.78, 2.01)	
**≥35,000**					
**Better**	775/127316	1.00	1.00	1.00	
**Same**	2462/384871	1.29 (1.18, 1.41)	1.29 (1.18, 1.40)	1.26 (1.15, 1.37)	
**Worse**	519/61798	1.94 (1.73, 2.18)	1.89 (1.68, 2.12)	1.76 (1.57, 1.98)	
**Cigarette smoking**					0.21
**Never**					
**Better**	2194/374639	1.00	1.00	1.00	
**Same**	9066/1361516	1.31 (1.25, 1.37)	1.30 (1.24, 1.36)	1.27 (1.21, 1.33)	
**Worse**	3170/330686	1.97 (1.86, 2.08)	1.91 (1.81, 2.02)	1.79 (1.69, 1.89)	
**Former**					
**Better**	383/38017	1.00	1.00	1.00	
**Same**	1208/111150	1.31 (1.16, 1.47)	1.30 (1.15, 1.46)	1.27 (1.12, 1.43)	
**Worse**	439/28689	2.06 (1.78, 2.37)	2.01 (1.73, 2.33)	1.90 (1.64, 2.21)	
**Current**					
**Better**	1461/237179	1.00	1.00	1.00	
**Same**	4665/699514	1.18 (1.11, 1.26)	1.18 (1.11, 1.26)	1.15 (1.08, 1.22)	
**Worse**	1439/141675	1.91 (1.77, 2.06)	1.88 (1.74, 2.03)	1.74 (1.61, 1.88)	
**Alcohol drinking**					0.006

**Never**					
**Better**	1430/226918	1.00	1.00	1.00	
**Same**	7371/1026479	1.25 (1.18, 1.33)	1.25 (1.18, 1.33)	1.22 (1.15, 1.29)	
**Worse**	2670/253196	1.81 (1.70, 1.94)	1.79 (1.68, 1.91)	1.67 (1.56, 1.78)	
**Former**					
**Better**	68/6707	1.00	1.00	1.00	
**Same**	359/29972	1.17 (0.89, 1.54)	1.19 (0.90, 1.56)	1.17 (0.89, 1.54)	
**Worse**	187/13611	1.76 (1.32, 2.35)	1.77 (1.32, 2.37)	1.76 (1.31, 2.36)	
**Current**					
**Better**	2540/416209	1.00	1.00	1.00	
**Same**	7209/1115730	1.26 (1.21, 1.32)	1.27 (1.21, 1.33)	1.23 (1.18, 1.29)	
**Worse**	2191/234245	2.06 (1.94, 2.18)	2.04 (1.93, 2.17)	1.91 (1.80, 2.03)	
**Physical activity**					0.11
**Low**					
**Better**	1914/182631	1.00	1.00	1.00	
**Same**	7196/656658	1.28 (1.21, 1.34)	1.27 (1.2, 1.34)	1.23 (1.17, 1.30)	
**Worse**	2703/170373	2.05 (1.93, 2.18)	2.00 (1.88, 2.12)	1.84 (1.73, 1.96)	
**Moderate**					
**Better**	1350/240532	1.00	1.00	1.00	
**Same**	4388/722107	1.27 (1.19, 1.35)	1.26 (1.18, 1.34)	1.24 (1.16, 1.32)	
**Worse**	1380/168379	1.91 (1.77, 2.06)	1.87 (1.73, 2.02)	1.75 (1.61, 1.89)	
**High**					

**Better**	774/226671	1.00	1.00	1.00	
**Same**	3355/793416	1.21 (1.12, 1.31)	1.21 (1.11, 1.31)	1.19 (1.10, 1.29)	
**Worse**	965/162299	1.72 (1.56, 1.90)	1.69 (1.53, 1.87)	1.63 (1.47, 1.80)	
**BMI**					0.21
**<18.5 kg/m^2**					
**Better**	107/15798	1.00	1.00	1.00	
**Same**	741/89954	1.38 (1.12, 1.71)	1.35 (1.09, 1.67)	1.34 (1.08, 1.65)	
**Worse**	387/33516	1.97 (1.58, 2.46)	1.85 (1.47, 2.31)	1.79 (1.42, 2.24)	
**18.5-23.9 kg/m^2**					
**Better**	1617/319765	1.00	1.00	1.00	
**Same**	6784/1168757	1.27 (1.20, 1.34)	1.26 (1.19, 1.34)	1.24 (1.17, 1.31)	
**Worse**	2262/266220	2.03 (1.90, 2.16)	1.98 (1.85, 2.11)	1.85 (1.73, 1.98)	
**24.0-27.9 kg/m^2**					
**Better**	1671/240369	1.00	1.00	1.00	
**Same**	5256/705365	1.24 (1.17, 1.31)	1.23 (1.16, 1.30)	1.20 (1.13, 1.27)	
**Worse**	1575/146361	1.90 (1.77, 2.05)	1.82 (1.70, 1.96)	1.69 (1.57, 1.82)	
**≥28.0 kg/m^2**					
**Better**	643/73903	1.00	1.00	1.00	
**Same**	2158/208105	1.33 (1.22, 1.46)	1.32 (1.21, 1.45)	1.29 (1.17, 1.41)	
**Worse**	824/54954	1.96 (1.76, 2.18)	1.90 (1.71, 2.12)	1.75 (1.57, 1.96)	
**History of diabetes**					0.58

**No**					
**Better**	3688/627060	1.00	1.00	1.00	
**Same**	13489/2073825	1.26 (1.21, 1.31)	1.26 (1.21, 1.30)	1.24 (1.19, 1.28)	
**Worse**	4177/456726	1.90 (1.82, 1.99)	1.87 (1.79, 1.96)	1.80 (1.71, 1.88)	
**Yes**					
**Better**	350/22775	1.00	1.00	1.00	
**Same**	1450/98355	1.18 (1.04, 1.33)	1.16 (1.03, 1.31)	1.15 (1.02, 1.30)	
**Worse**	871/44325	1.72 (1.51, 1.95)	1.65 (1.45, 1.88)	1.60 (1.41, 1.83)	
**History of hypertension**					0.67
**No**					
**Better**	2110/461306	1.00	1.00	1.00	
**Same**	7229/1476027	1.26 (1.2, 1.33)	1.26 (1.2, 1.33)	1.25 (1.18, 1.31)	
**Worse**	2262/328713	1.9 (1.79, 2.02)	1.87 (1.76, 1.99)	1.79 (1.68, 1.91)	
**Yes**					
**Better**	1928/188528	1.00	1.00	1.00	
**Same**	7710/696154	1.23 (1.17, 1.30)	1.22 (1.16, 1.29)	1.21 (1.15, 1.28)	
**Worse**	2786/172338	1.89 (1.78, 2.01)	1.83 (1.72, 1.94)	1.77 (1.66, 1.88)	

*Model 1: adjusted for age, marital status, education level, household income, occupation, and menopausal status;

†Model 2: model 1 plus sleep problems, cigarette smoking, alcohol drinking, physical activity, body mass index, and family history of heart disease;

‡Model 3: model 2 plus baseline major depression, diabetes, hypertension and other comorbidities.

Table S5. Risk of Ischemic Heart Disease According to Two Measures of Self-rated Health Status

Exposures	A* HR (95% CI)	B† HR (95% CI)	C‡ HR (95% CI)
**Global self-rated health (Reference=Excellent)**			
**Good**	1.02 (0.98, 1.07)		0.98 (0.94, 1.03)
**Fair**	1.32 (1.27, 1.37)		1.19 (1.14, 1.25)
**Poor**	1.76 (1.68, 1.85)		1.37 (1.29, 1.45)
**Age-comparative self-rated health (Reference=Better)§**			
**Same**		1.23 (1.19, 1.27)	1.14 (1.10, 1.19)
**Worse**		1.78 (1.70, 1.86)	1.46 (1.37, 1.53)
**Don't know**		1.46 (1.34, 1.59)	1.27 (1.17, 1.39)

* only global self-rated health was included in model 3 (adjusted for age, marital status, education level, household income, occupation, and menopausal status, sleep problems, cigarette smoking, alcohol drinking, physical activity, body mass index, family history of heart disease, baseline major depression, diabetes, hypertension, and other comorbidities).

† only age-comparative self-rated health was included in model 3.

‡ both global self-rated health and age-comparative self-rated health were included in model 3.

§ Participants who reported “don’t know” to the age-comparative self-rated health question were not excluded in this analysis.

Table S6. The Distribution of Global Self-rated Health Status by Age-comparative Self-rated Health Status

Global self-rated health	Age-comparative self-rated health, N (%)				Total, N (%)
	Better	Same	Worse	Don't Know	
**Excellent**	46 875 (51.7)	39414 (12.8)	717 (1.0)	1334 (8.9)	88340 (18.2)
**Good**	30 651 (33.8)	105 831 (34.2)	2 862 (4.0)	1678 (11.2)	141022 (29.0)
**Fair**	12 320 (13.6)	157 926 (51.1)	31 521 (43.9)	10391 (69.3)	212158 (43.6)
**Poor**	892 (1.0)	5851 (1.9)	36 691 (51.1)	1587 (10.6)	45021 (9.3)
**Total, N (%)**	90 738 (18.6)	309 022 (63.5)	71791 (14.8)	14990 (3.1)	486541

Table S7. Sensitivity Analysis: Risk of Ischemic Heart Disease According to Two Measures of Self-rated Health Status

**Excluding participants with baseline conditions**	**Major depression (n=2,972)**	**Baseline comorbidities (n=238,007)**	**Died or developed IHD in the first 2 years (n=9,147)**
	**HR (95% CI)**	**HR (95% CI)**	**HR (95% CI)**
**Global self-rated health**			
**Excellent**	1.00	1.00	1.00
**Good**	1.02 (0.98, 1.07)	1.05 (0.98, 1.13)	1.06 (1.01, 1.11)
**Fair**	1.32 (1.27, 1.37)	1.32 (1.24, 1.41)	1.32 (1.26, 1.37)
**Poor**	1.78 (1.69, 1.87)	1.88 (1.72, 2.06)	1.76 (1.66, 1.86)
**Age-comparative self-rated health**			
**Better**	1.00	1.00	1.00
**Same**	1.23 (1.19, 1.27)	1.26 (1.18, 1.33)	1.22 (1.17, 1.27)
**Worse**	1.79 (1.71, 1.87)	1.89 (1.74, 2.04)	1.75 (1.67, 1.84)

Hazards ratios (95% confidence interval) were calculated after adjustment for age, marital status, education level, household income, occupation, and menopausal status, sleep problems, cigarette smoking, alcohol drinking, physical activity, body mass index, family history of heart disease, baseline major depression, diabetes, hypertension, and other comorbidities.

Figure S1. Risk of Ischemic Heart Disease According to global Self-rated Health Status by study locations

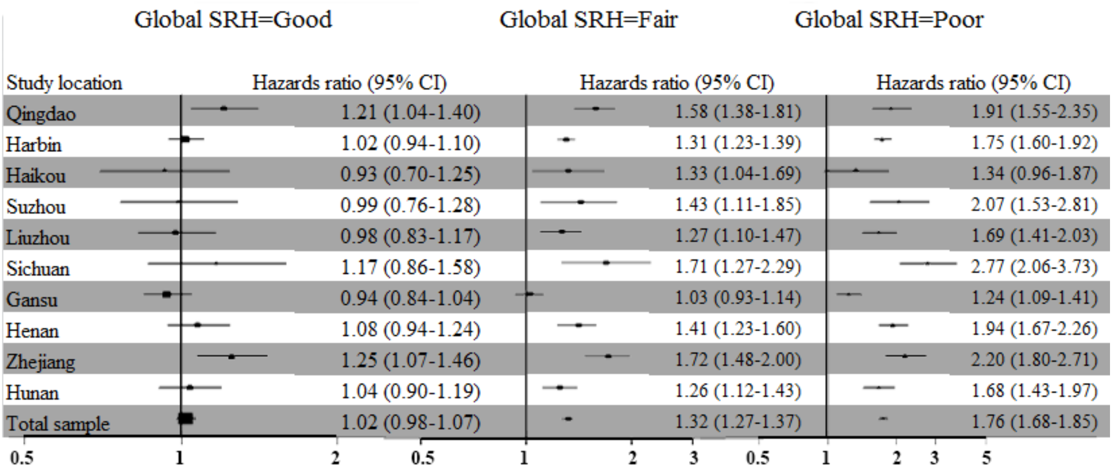

Hazards ratios (95% CI) were calculated after adjustment for age, marital status, education level, household income, occupation, and menopausal status, sleep problems, cigarette smoking, alcohol drinking, physical activity, body mass index, family history of heart disease, and baseline major depression, diabetes, hypertension and other comorbidities.

Figure S2. Risk of Ischemic Heart Disease According to Age-comparative Self-rated Health Status by study locations

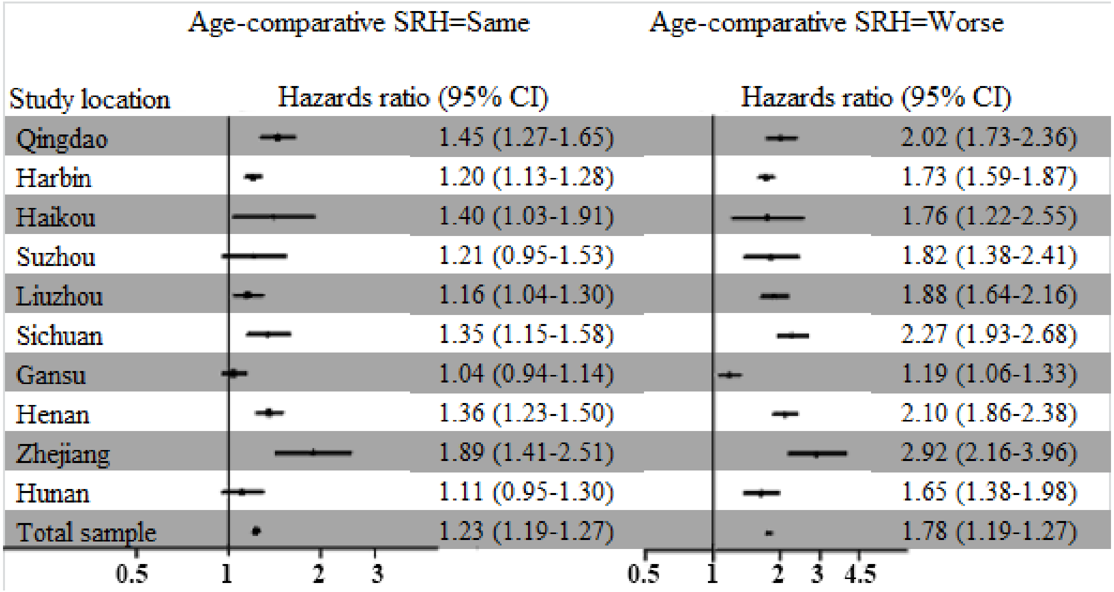

Hazards ratios (95% CI) were calculated after adjustment for age, marital status, education level, household income, occupation, and menopausal status, sleep problems, cigarette smoking, alcohol drinking, physical activity, body mass index, family history of heart disease, and baseline major depression, diabetes, hypertension and other comorbidities.
